# Supplementary material for: A pharmacoproteomic landscape of organotypic intervention responses in Gram-negative sepsis
Source: Nat Commun. 2023 Jun 17;14:3603. doi: 10.1038/s41467-023-39269-9 (PMC10276868; doi:10.1038/s41467-023-39269-9)
Supplement: Supplementary file 1 — Supplementary Information [file 41467_2023_39269_MOESM1_ESM.pdf]

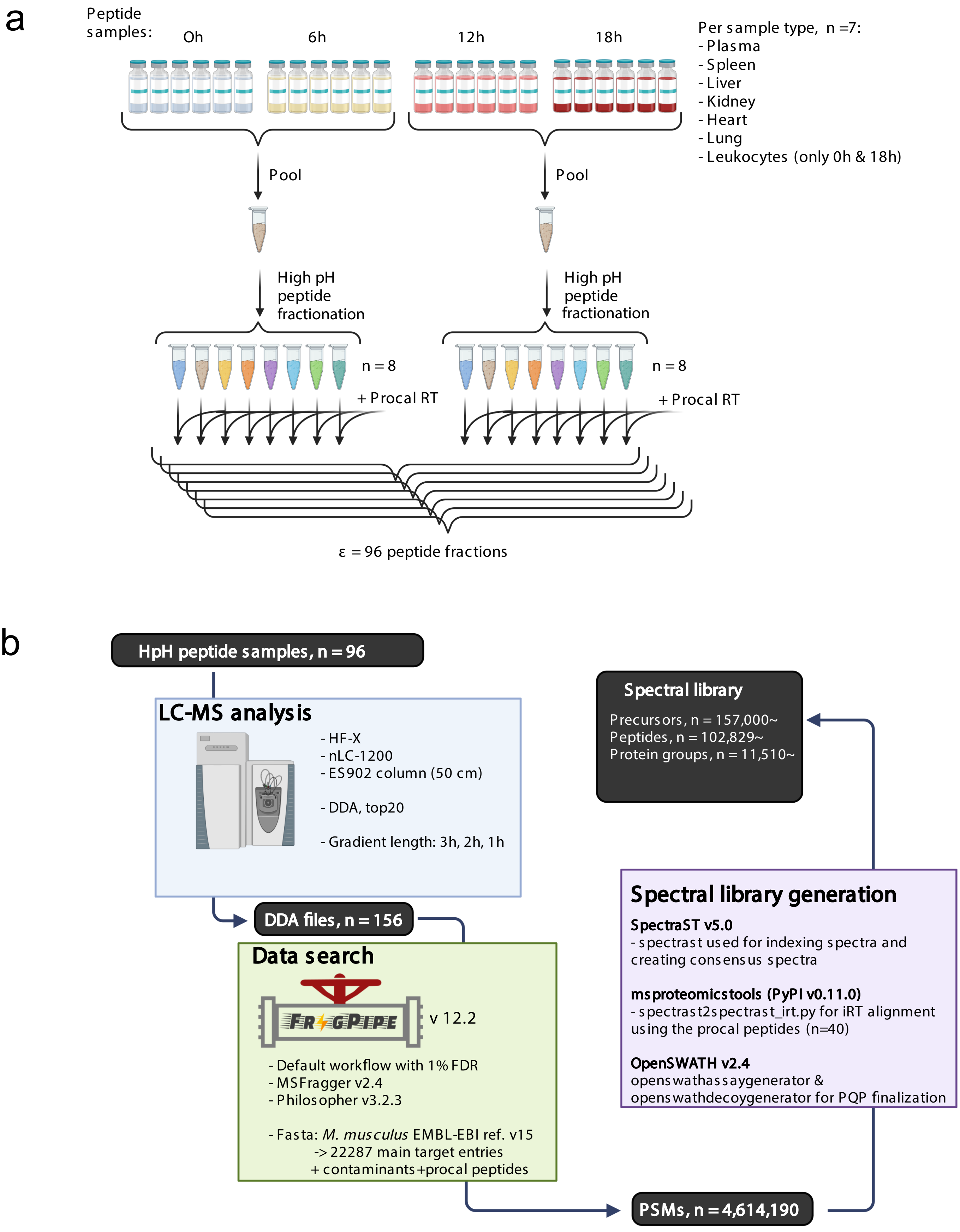

Supplementary Figure 1 Outline of spectral library generation
a)Organs homogenates, leukocytes and plasma were pooled and fractionated using high pH peptide fractionation (Pierce), spiked with retention time peptides (Procal RT)
b)LC MS analysis and data searching and creation of spectral library creation (See methods for details)
Image was created with bioRender (biorender.com).

a

180805	Heart	Kidney	Liver	Lungs	Plasma	Spleen
Naive	5	5	5	5	4	5
Inf_6h	6	6	6	6	6	6
Inf_12h	6	6	6	6	5	6
Inf_18h	6	6	6	6	6	6

191210	Heart	Kidney	Leukocytes	Liver	Plasma	Spleen
Naive	6	6	5	6	6	6
Inf_18h	6	6	5	6	6	6
Inf_Mem8h	6	6	5	6	5	6
Inf_Gcc8h	7	7	7	7	7	6
Inf_Gcc2h	6	6	5	6	5	6
Inf_Gcc2h+Mem8h	7	7	6	7	6	7
Inf_Gcc8h+Mem8h	6	6	5	6	6	6

200319	Kidney	Leukocytes	Plasma	Spleen
Naive	6	5	6	6
Inf_18h	3	3	3	3
Inf_Mem2h	5	5	5	5
Inf_Mem8h	5	5	5	5
Inf_Gcc8h	5	5	5	5
Inf_Gcc8h+Mem8h	5	5	5	5

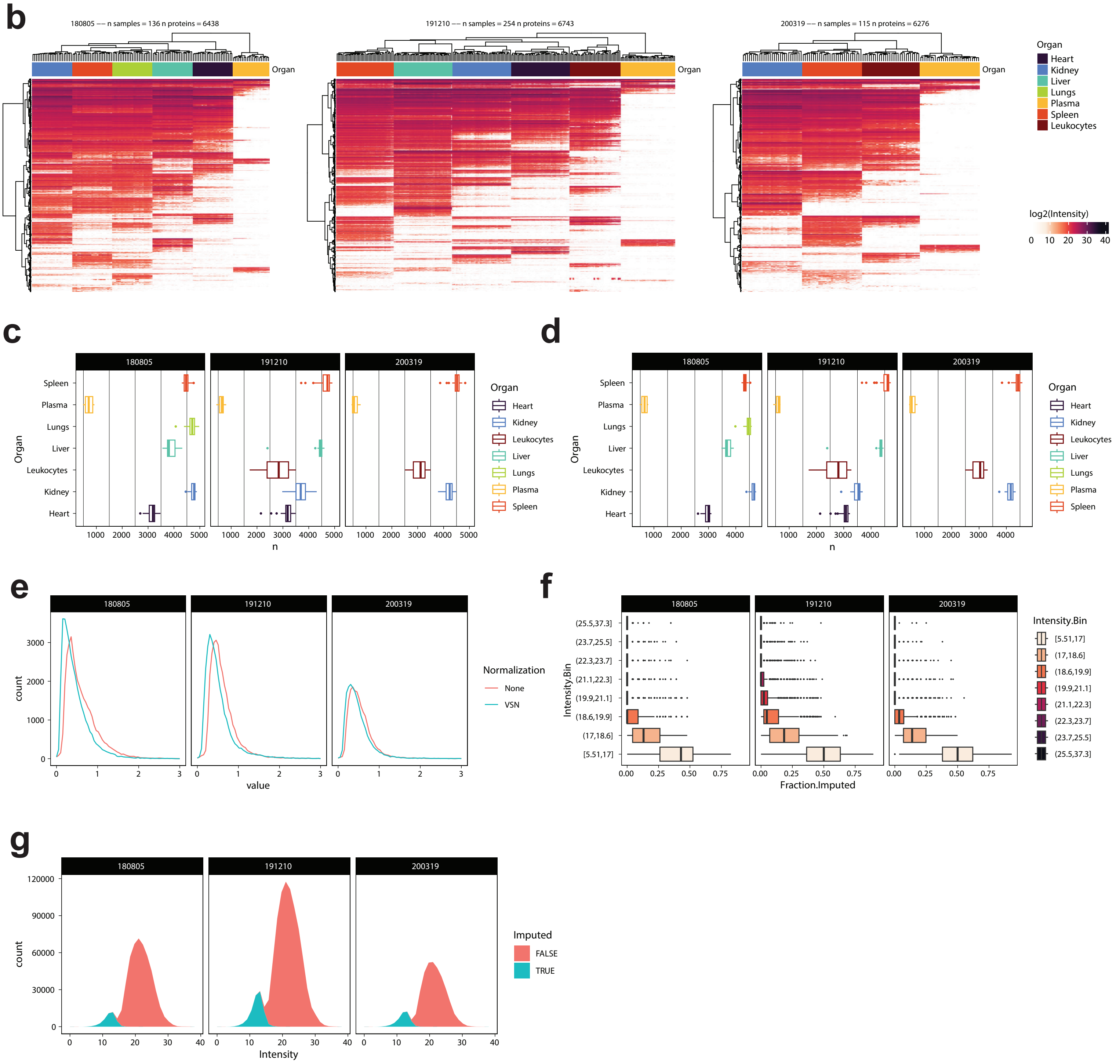

Supplementary Figure 2. Quality control and preprocessing of DIA data. The DIA data is divided into three datasets – 180805 sepsis time course, 191210 & 200319 interventions. a) The sample groups and replicate count of each experiment and group. b) Heatmaps of protein intensity across experiments. c) Protein ID count per sample of unfiltered data d) Protein ID count per sample after applying missing value filter (the missing value filter requires at most 1 missing value in 1 sample group). e) Distribution of coefficient of variation (CV) of protein intensities per organ. Color indicates CV of raw intensities and CV of intensities after applying variance stabilizing normalization (VSN). f) Median proteins intensities per experi-ment & organ are discretised into 8 categories, y-axis Intensity.Bin, and the fraction of missing values per protein and organ boxplots are on the x-axis Fraction.Imputed. g) Intensity distribution of measured and imputed protein intensities per experiment. Box boundaries represent first and third quartiles, center line indicates median values. The upper whisker extends from the hinge to the largest value no further than 1.5 * IQR from the hinge (where IQR is the inter-quartile range, or distance between the first and third quartiles). The lower whisker extends from the hinge to the smallest value, at most 1.5 * IQR of the hinge. Data beyond the end of the whiskers are called "outlying" points and are plotted individually.

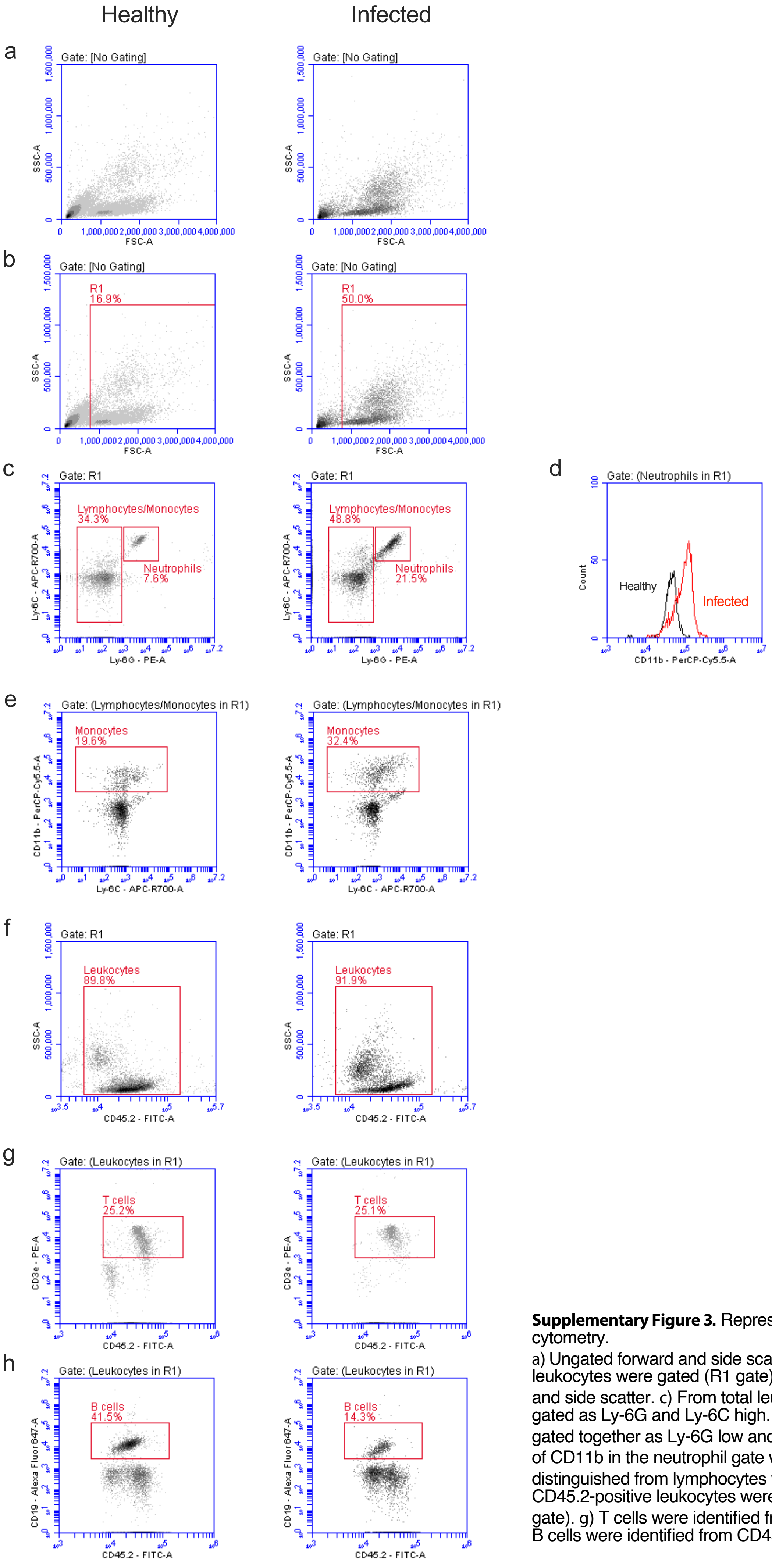

Supplementary Figure 3. Representative gating strategies for flow cytometry.

a) Ungated forward and side scatter plot. b) In the first panel, total leukocytes were gated (R1 gate) according to characteristic forward and side scatter. c) From total leukocytes (R1 gate), neutrophils were gated as Ly-6G and Ly-6C high. Lymphocytes and monocytes were gated together as Ly-6G low and Ly-6C intermediate/high. d) The MFI of CD11b in the neutrophil gate was determined. e) Monocytes were distinguished from lymphocytes with CD11b. f) In the second panel, CD45.2-positive leukocytes were gated from total leukocytes (R1 gate). g) T cells were identified from CD45.2-leukocytes with CD3e. h) B cells were identified from CD45.2-leukocytes with CD19.

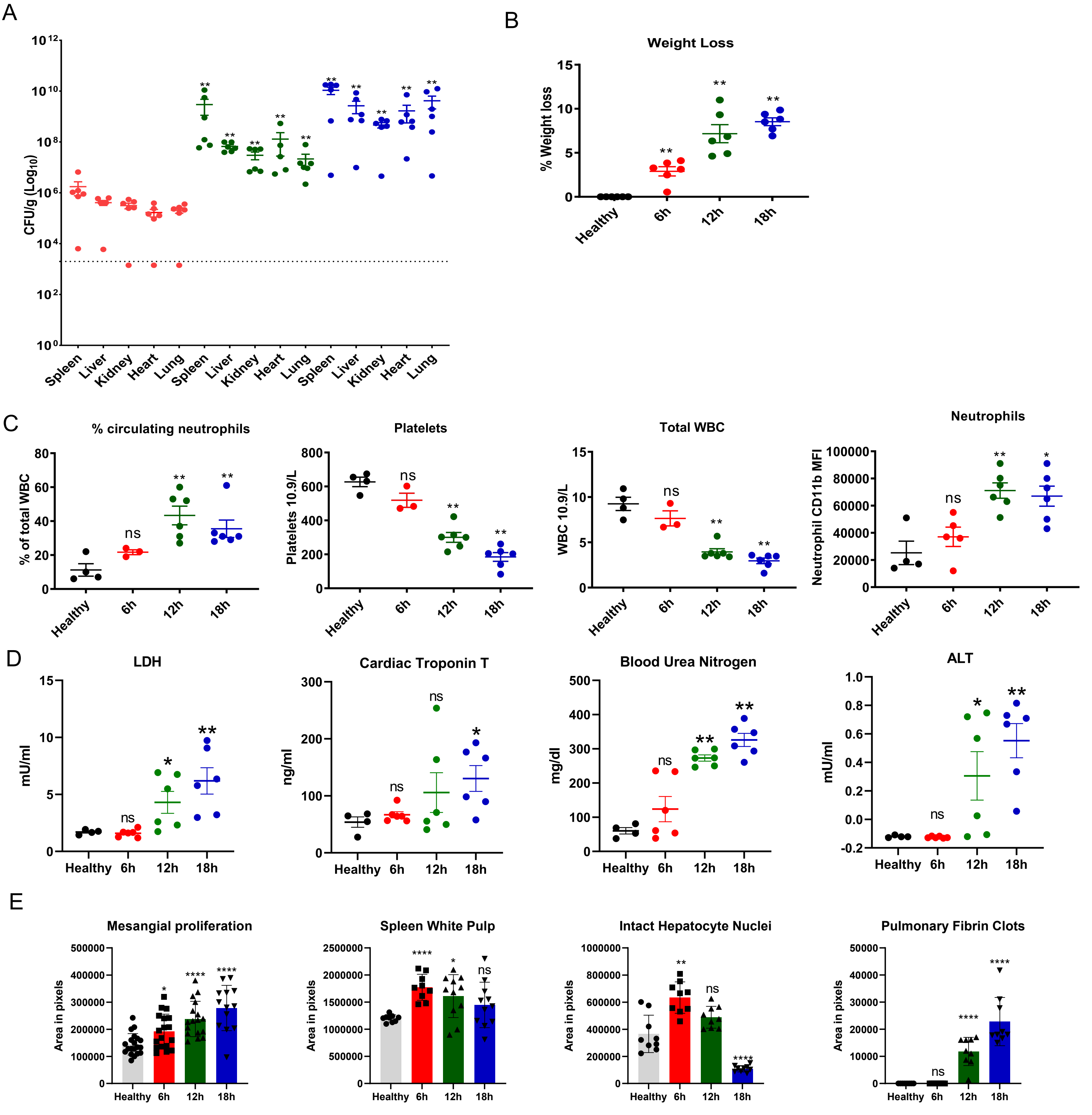

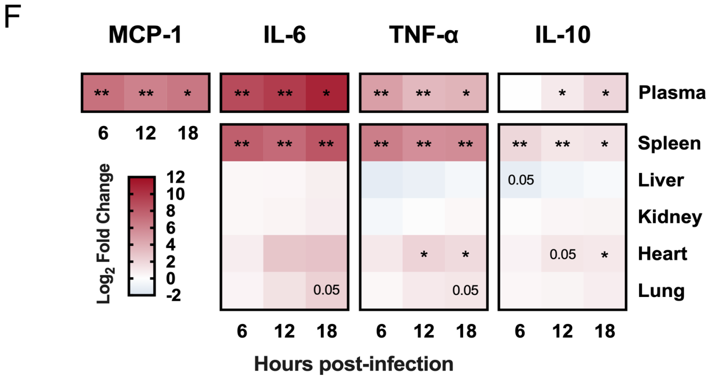

Supplementary Figure 4: Metadata for time course experiments a) Bacterial load b) Weight loss c) Circulating immune cell profile d) Organ damage markers in plasma e) Microscopic quantification of histological features f) Heatmap depicting Log2 Fold change of cytokines in plasma and organs. For bacterial load, cytokines, circulating immune cell profile, Organ damage markers in plasma, and heatmap depicting Log2 Fold change of cytokines in plasma and organs, all groups were compared to 0h by two-tailed Mann-Whitney, ****p < 0.0001; ***p < 0.001; **p < 0.01; *p < 0.05; ns, not significant and wherever applicable, bars indicate mean and error bars depict SEM of the mean. For the microscopic quantification, Bars represent mean, dots represent individual values from 3 animals per time point (n=3) and error bars represent SEM of the mean. All conditions were compared with healthy (0 h) using Kruskal-Wallis with *** p<0.0005, ** p<0.005, * p<0.05 and ns=non-significant.

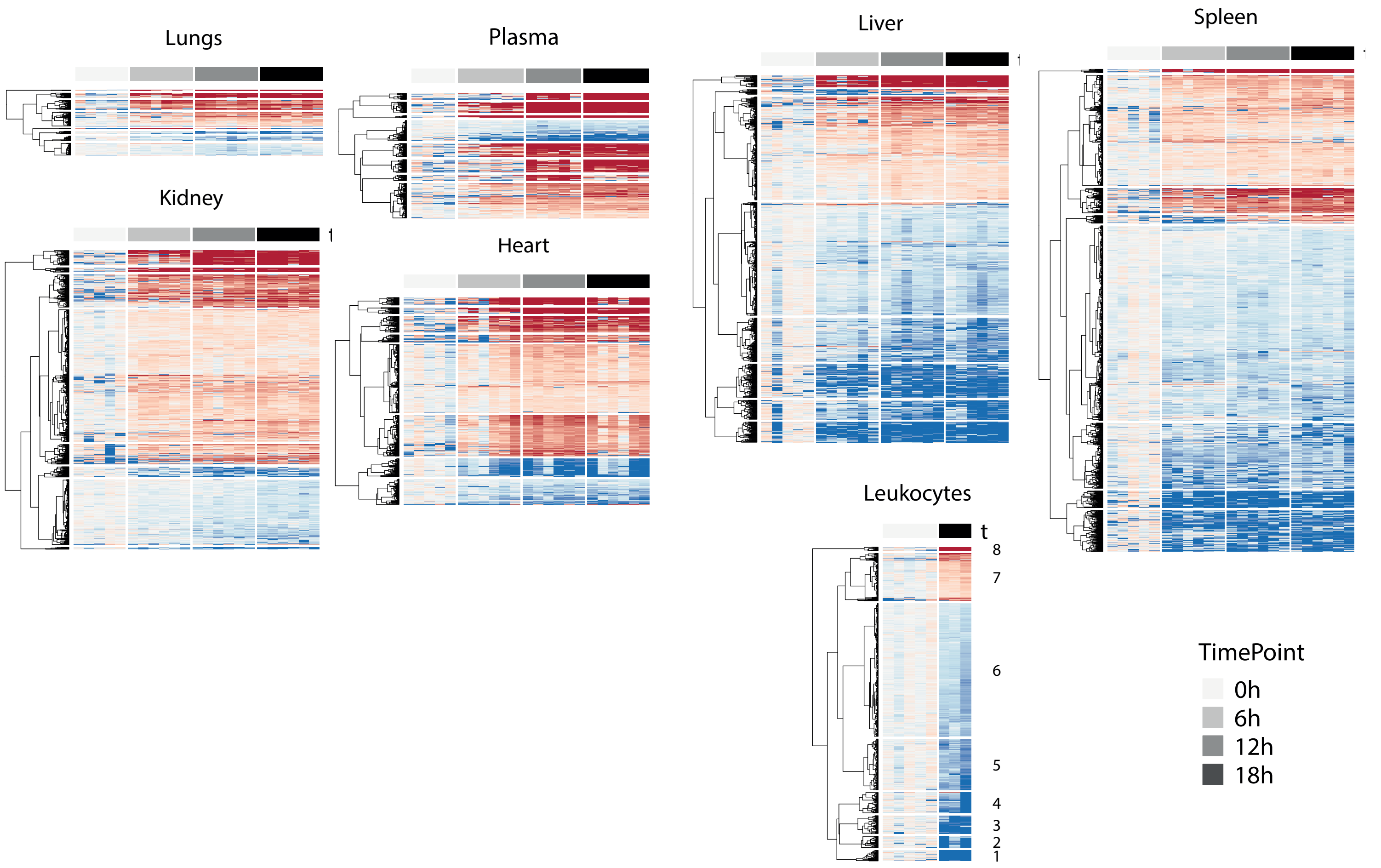

Supplementary Figure 5: Heatmaps depicting all DAPs observed in organs, plasma and leukocytes during the time course of sepsis.

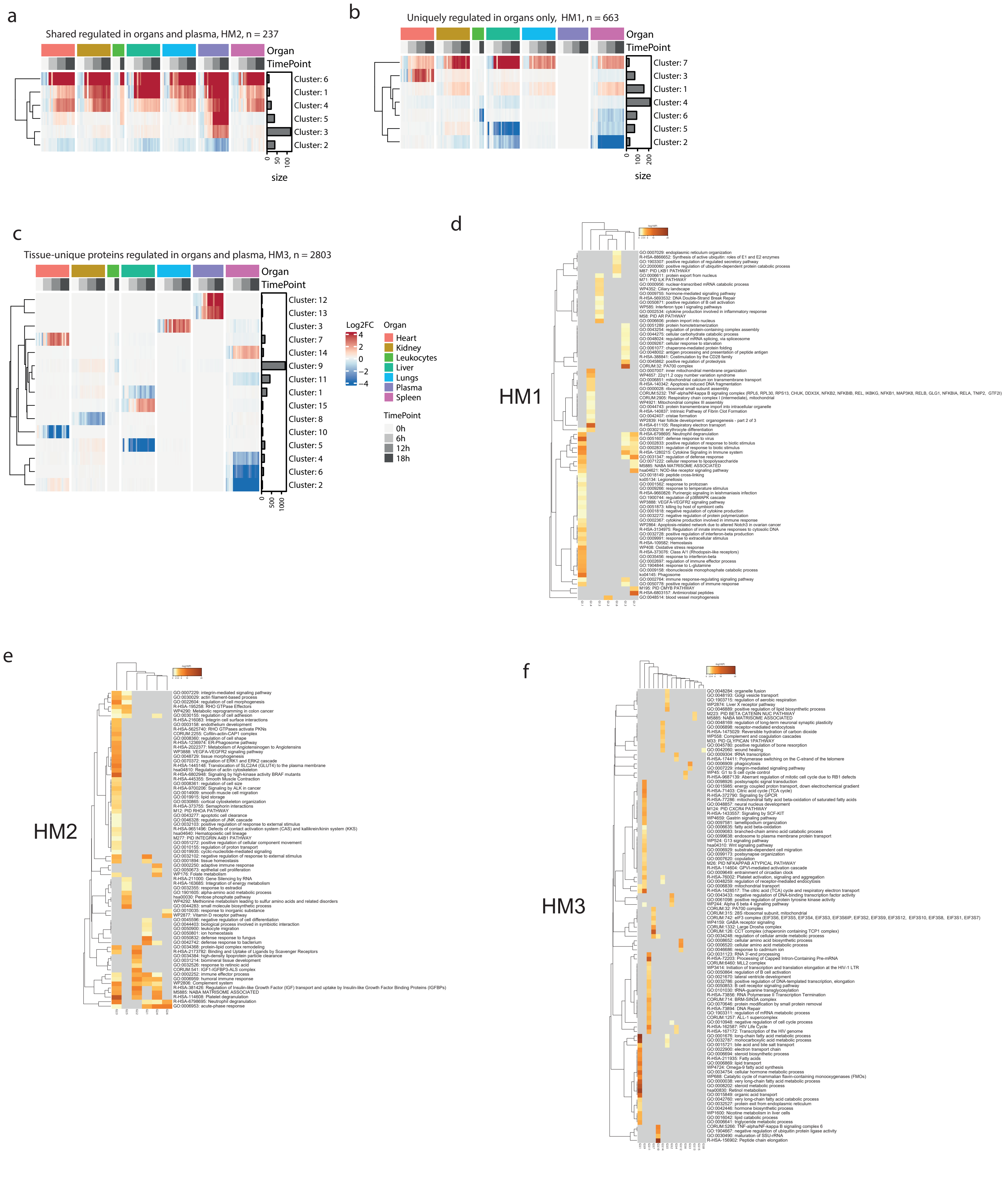

Supplementary Figure 6 Regulation of DAPs in organs, plasma and leukocytes
a) HM2, Shared DAPs regulated in atleast 1 organ, plasma and leukocytes.
b) HM1, Regulated DAPs shared across organs but not plasma.
c) HM3, Regulated DAPs shared across organs and plasma.
d) Metascape GO terms of cluster HM1, tissue unique DAPs upregulated in organs only.
e) Metascape terms of cluster HM2, shared regulated in organs and plasma
f) Metascape terms for tissue-unique proteins regulated in organs and plasma, HM3.

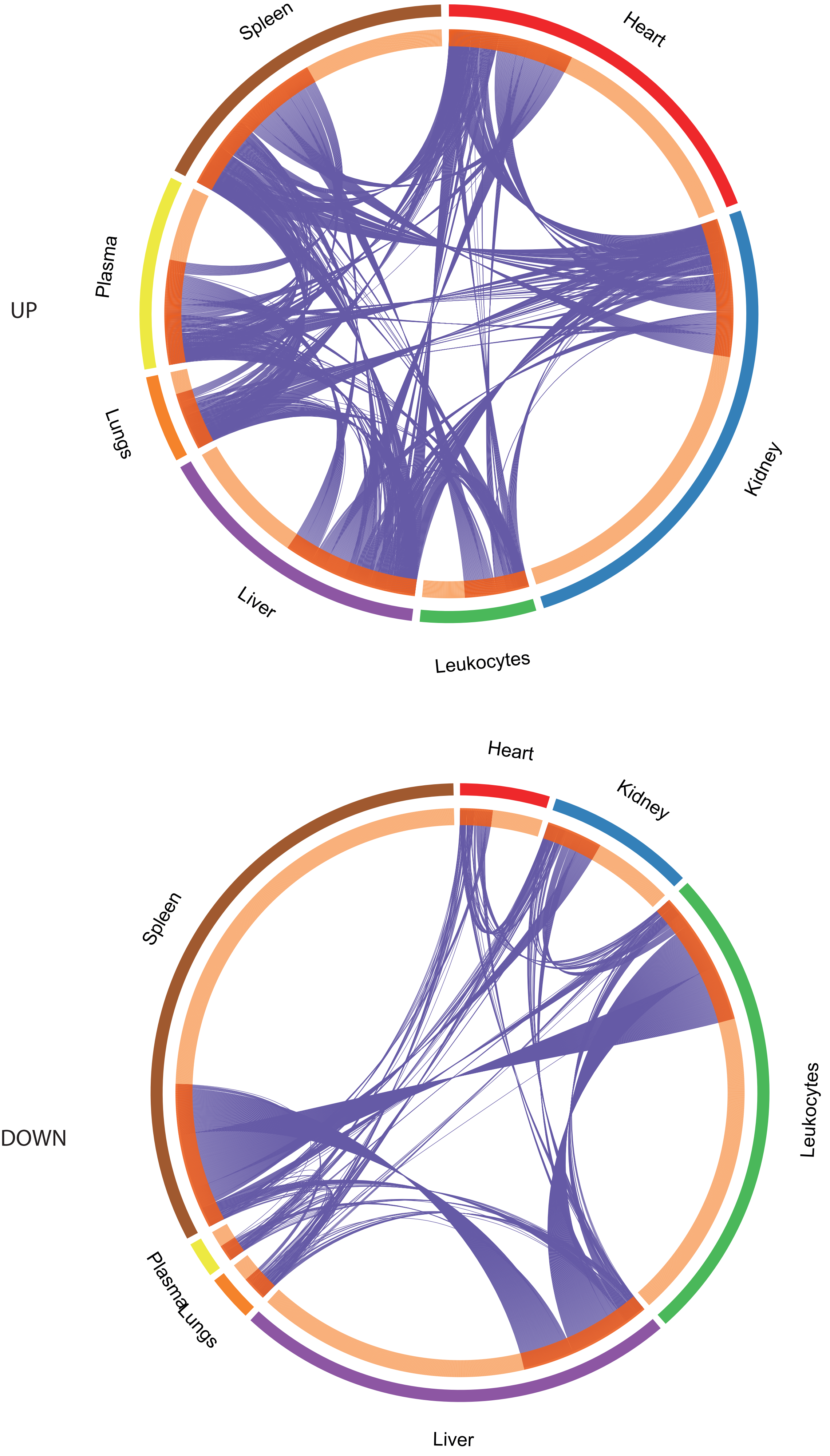

Supplementary Figure 7 Circosplots of GO terms and regulated DAPs in organs, leukocytes and plasma.

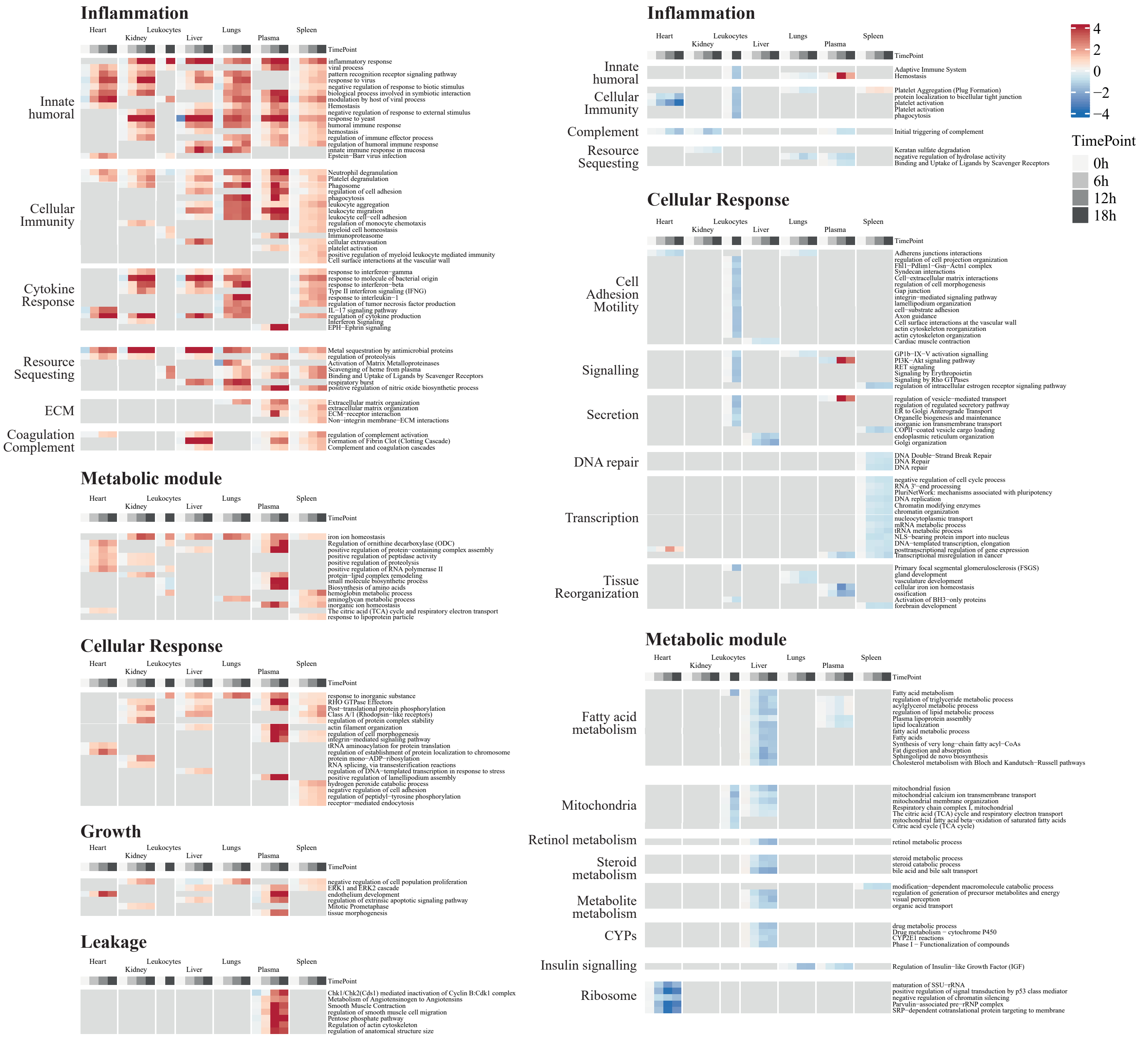

Supplementary Figure 8: Manually curated heatmap of metascape GO terms depicting up- and down-regulated terms during the time course.

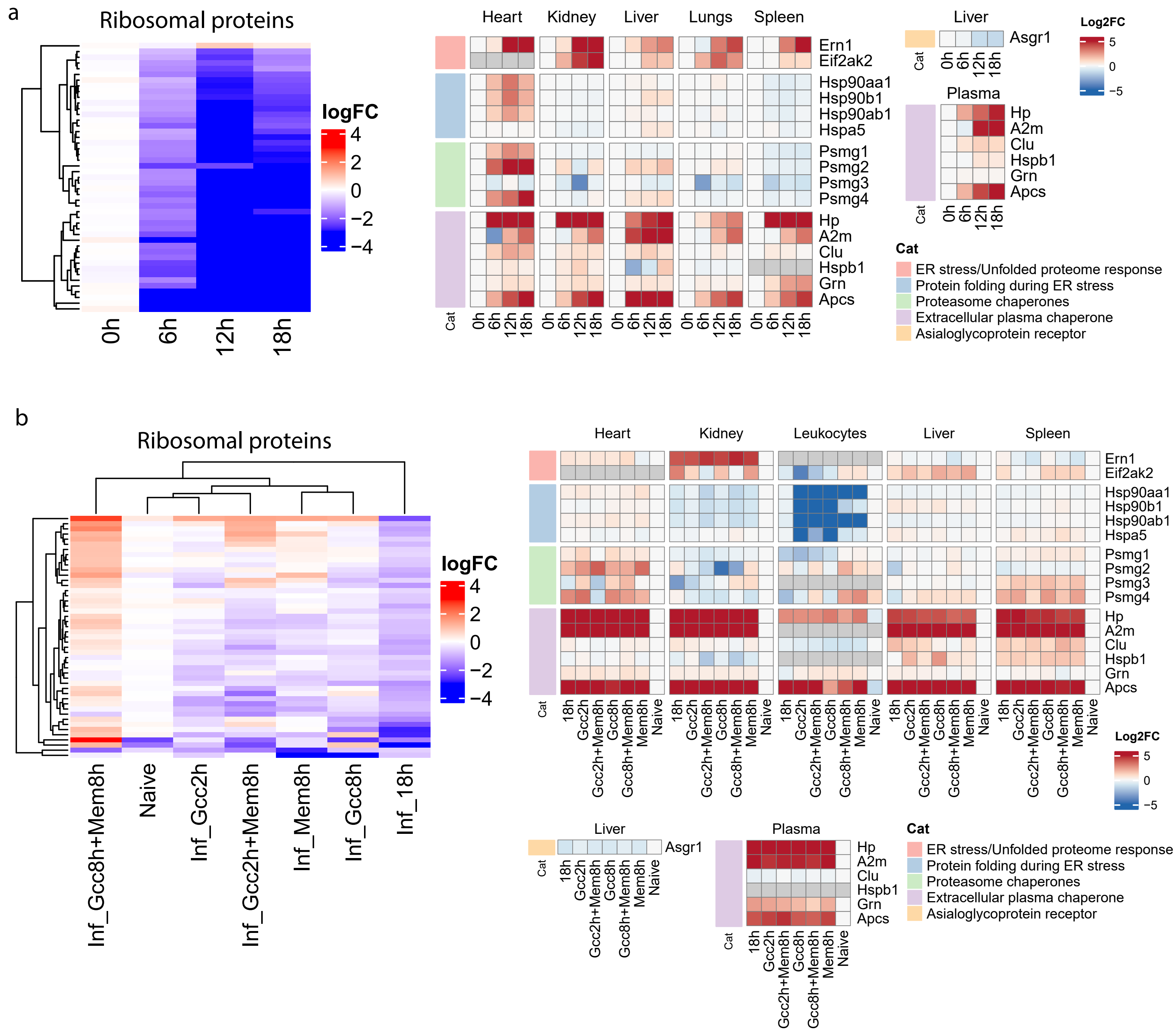

Supplementary Figure 9 Mechanisms of proteostasis in sepsis.

We observed a major reduction of cytosolic ribosomal components in the heart that constitute the first step of proteostasis. Our analysis also revealed a loss of proteostasis with progression of sepsis. Molecular sensors for detection of unfolded proteins in the endoplasmic reticulum like Inositol-requiring protein 1 (IRE1a) and interferon-induced double-stranded RNA-activated protein kinase (EIF2AK2), were up in all organs. Also, different isoforms of HSP90 (HSP90aa1, HSP90b1 and HSP90ab1) that are responsible for protein folding, and proteasome assembly chaperones (PSMG1-4) that promote the assembly of the 20S proteasome were elevated. In order to protect the secreted proteome, several extracellular chaperones like haptoglobin (HP), alpha-2-macroglobulin (A2M), clusterin (CLUS), heat shock protein beta-1 (HSPB1), progranulin (GRN), and serum amyloid P-component (APCS) in plasma and tissues were induced and could represent an active countermeasure for the formation of amyloid fibrils. The levels of ASGR1 responsible for clearance of asialylated proteins were downregulated in the liver during sepsis. In summary, the heart was strongly enriched for markers of perturbed proteostasis compared to others.
Panel a) shows these markers in the time course experiments and b) shows the levels in intervention experiments.

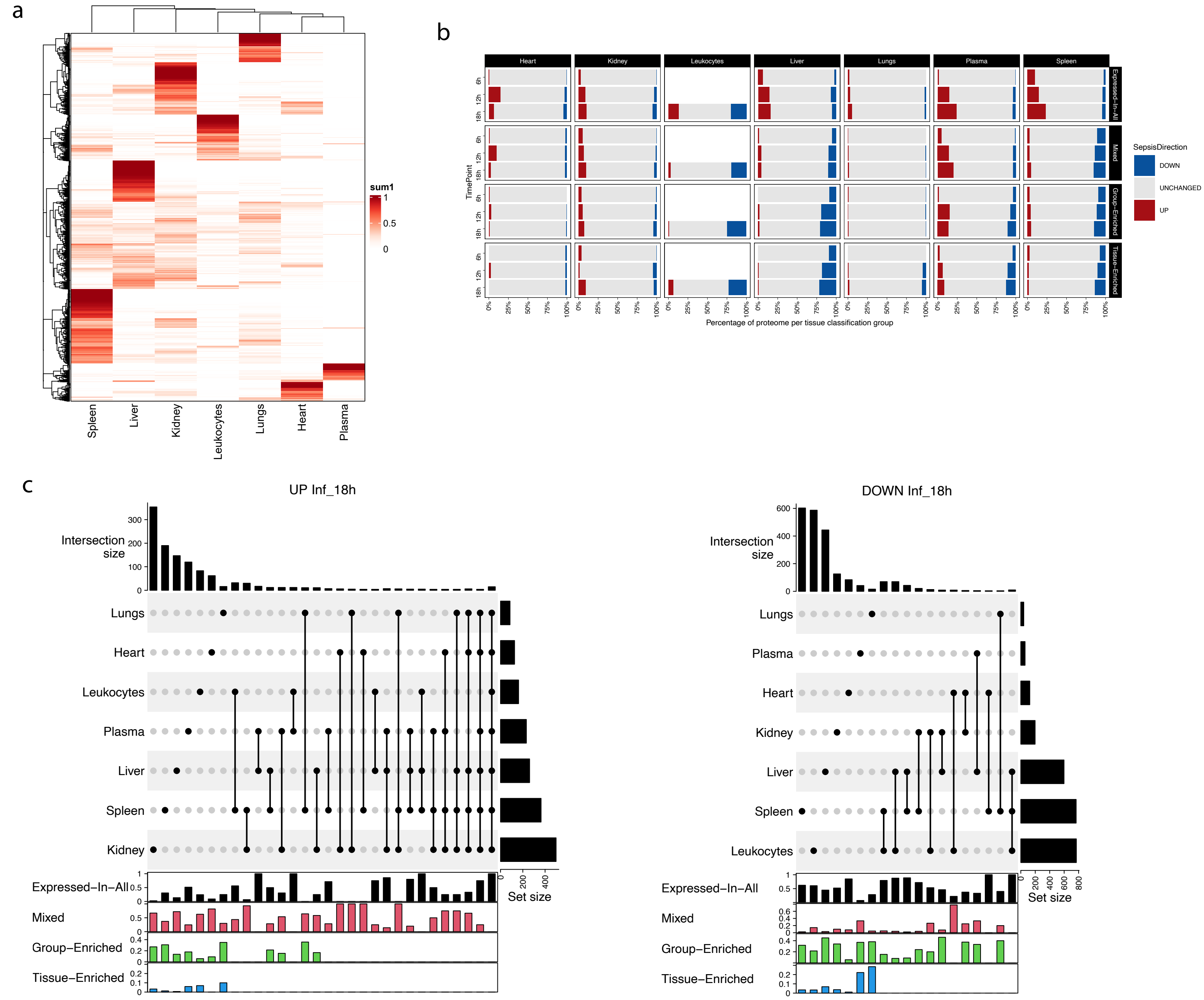

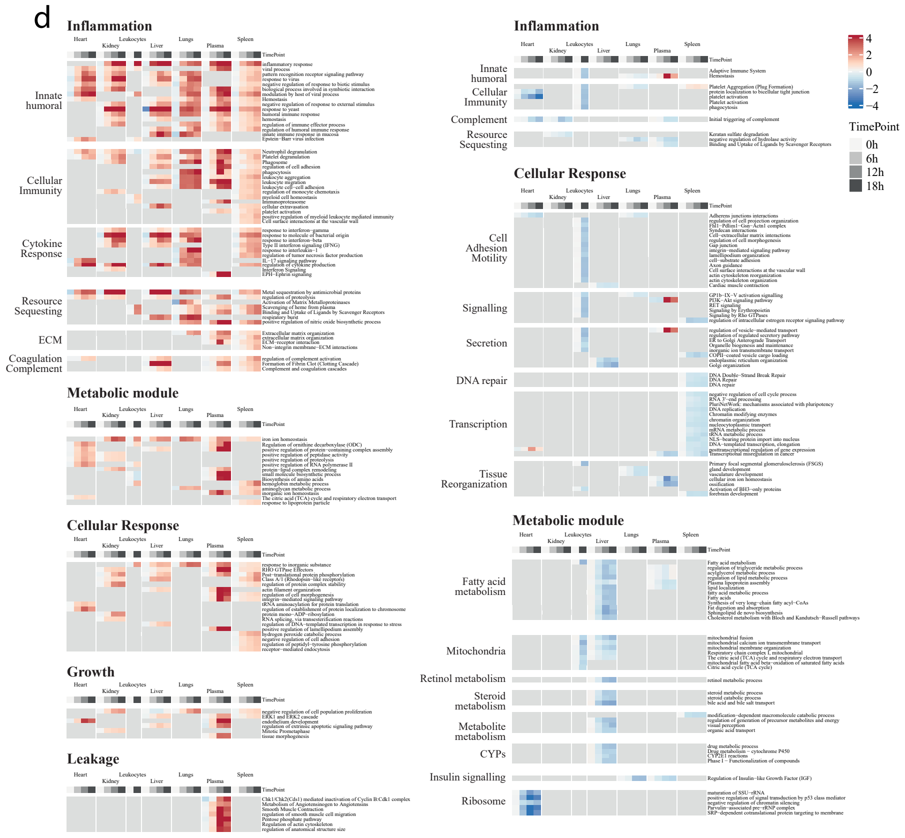

Supplementary Figure 10 Alterable proteome in sepsis.

a)Firstly, enrichment of tissue-specific genes was calculated using the TissueEnrich package by using control uninfected animals as the input. The teGeneRetrieval function is used to define tissue-specific genes, using the algorithm from the HPA (Uhlén et al. 2015). Proteins could be broadly categorised as expressed-in-all, mixed, group-enriched, and tissue-enriched. The heatmap depicts in panel A depicts organ specific proteins within organs.

b) Stacked bar graphs showing the percentages of up, down, and unchanged DAPs in organs. Most of the proteins that were upregulated belonged to the expressed-in-all category (blue), with spleen and plasma showing the highest level of enrichment. Whereas the majority of the downregulated proteins belonged to the tissue- and group-enriched (red) with liver and leukocytes showing the highest levels of downregulations (panel B). c) UpSet plots showing the complexities of the regulation and distribution of DAPs, and relationship between the organs. Each dot depicts DAP groups, and the lines show shared DAPs across 2 or more organs. Hence, individual unconnected dots in the plot depict uniquely upregulated DAPs in an organ. The intersect size at the top denotes the number of DAPs per dot and the set size denotes the sharing of DAPs between organs. The bar plot at the bottom of the data shows a breakdown of the DAPs contained within each dot into the 4 categories of expressed-in-all (black bars), mixed (red bars), group-enriched (green bars), and tissue-enriched (blue bars). The organs are then ordered from smallest to the largest (top to bottom) in terms of numbers of proteins and shared DAPs. Moving left across the plot shows uniquely regulated DAPs (represented by individual dots) in organs and the opposite direction shows side of the plot contained shared DAPs. d) A manually curated heatmap showing some of the interesting GO processes that we observe while sepsis. This was constructed by assigning GO terms to the up and down regulated DAPs in our data. The upregulated GO terms were associated were inflammation associated and were seen in all organs e.g., neutrophil degranulation in all organs. Many of the downregulated processes were tissue specific e.g., the downregulation of fatty acid metabolism in the liver. We have now added this in the figure for the ease of contextualizing all our findings.

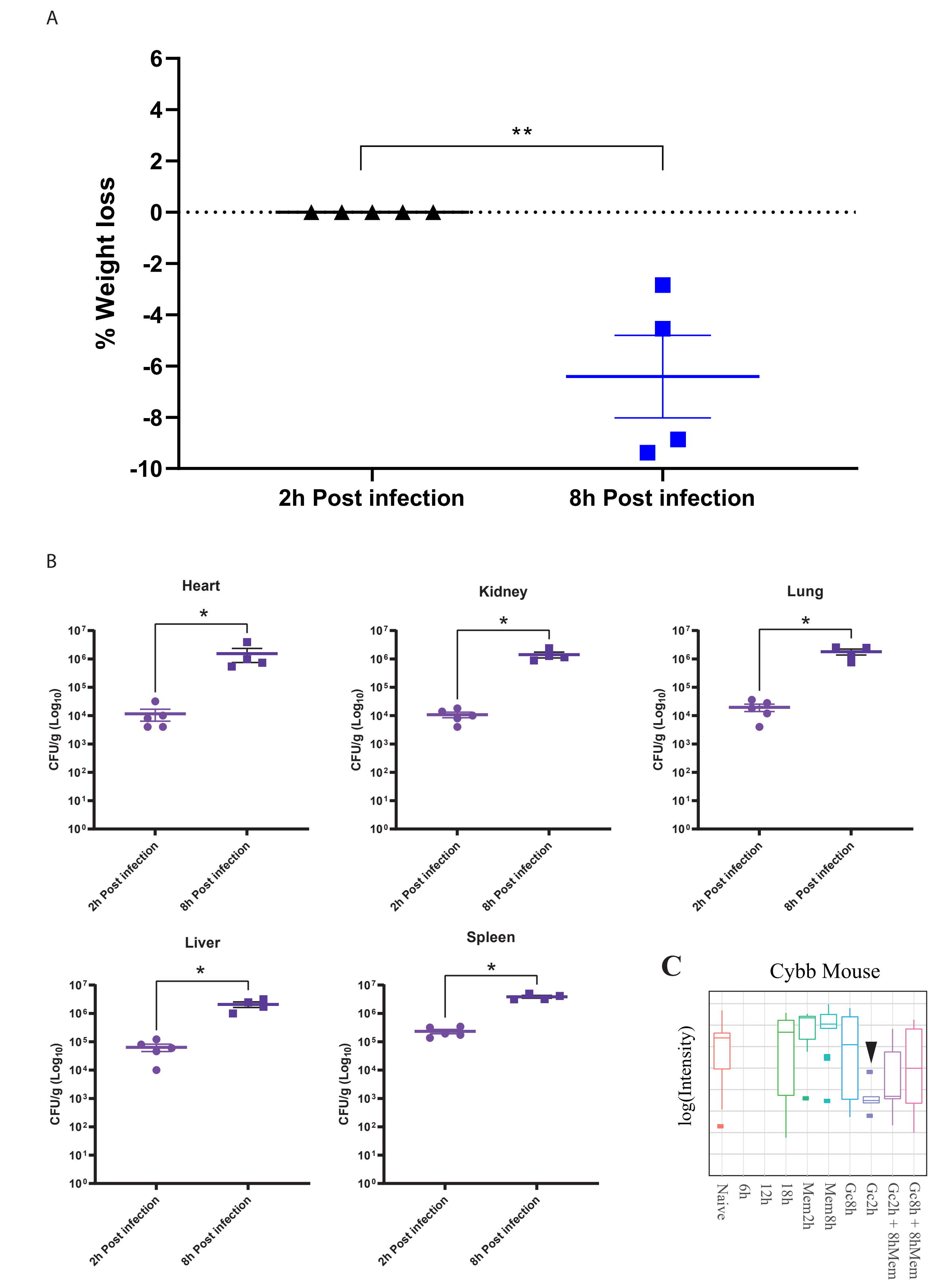

Supplementary figure Figure 11: Bacterial load 2h and 8h post-inoculation of bacteria
A) Weight loss B) Bacterial load C) Cybb log intensity levels in leukocytes in the treatment cohort. Data are presented as mean and error bars indicate SEM of mean. 8h.p.i mice were compared to 2 h.p.i mice by two-tailed Mann-Whitney, *** $p<0.0005$, ** $p<0.005$, * $p<0.05$ and ns=non-significant. Box boundaries represent first and third quartiles, center line indicates median values. The upper whisker extends from the hinge to the largest value no further than 1.5 * IQR from the hinge (where IQR is the inter-quartile range, or distance between the first and third quartiles). The lower whisker extends from the hinge to the smallest value, at most 1.5 * IQR of the hinge. Data beyond the end of the whiskers are called "outlying" points and are plotted individually.

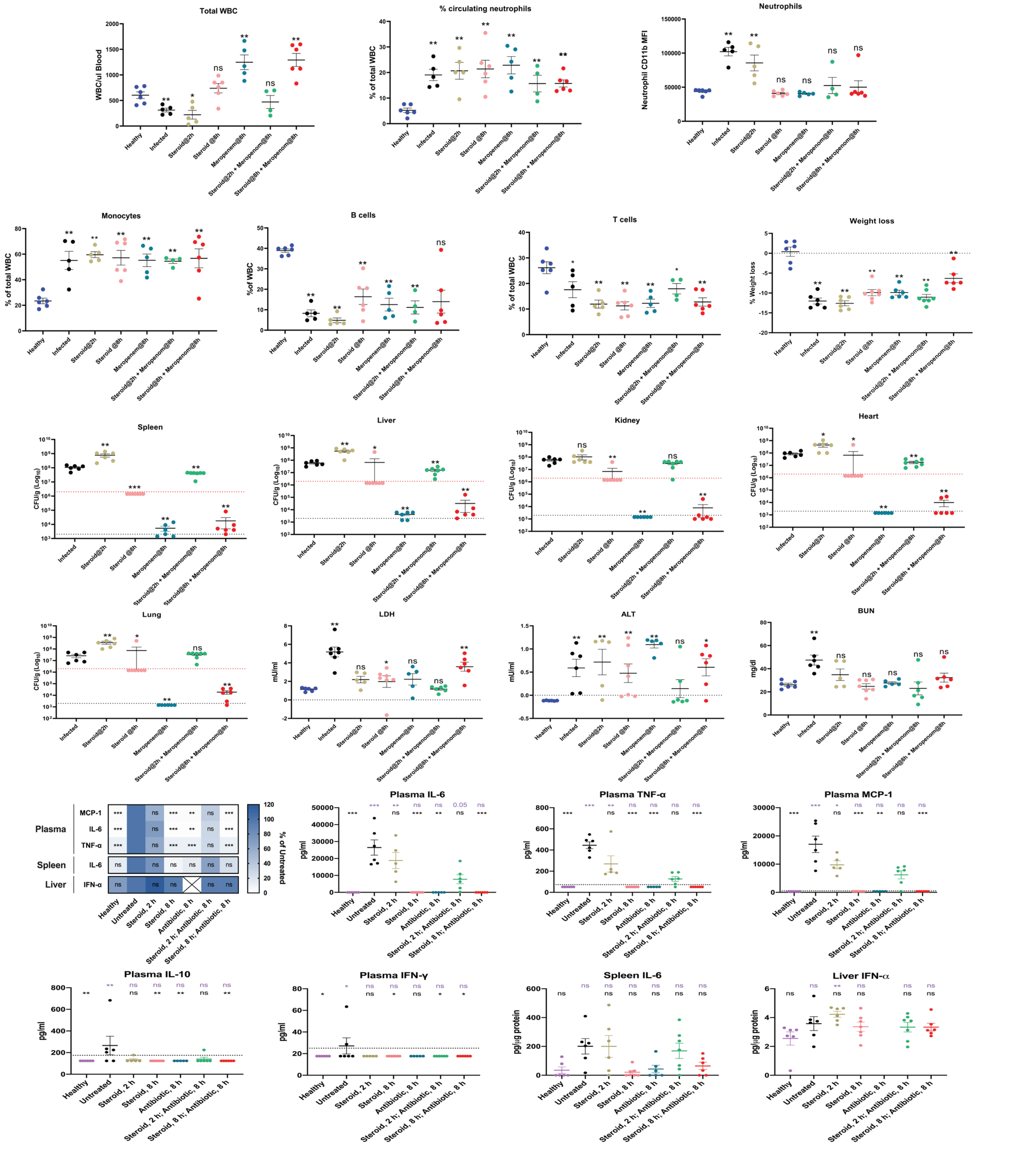

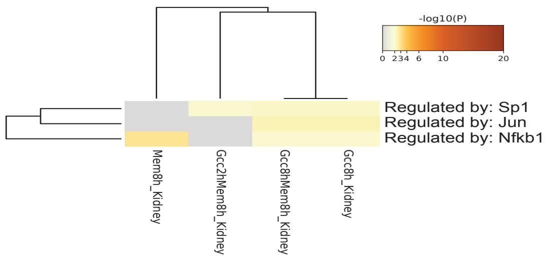

Supplementary Figure 12 Metadata of the treatment cohort
Physiological parameters of circulating immune cells, organ bacterial load, weightloss, organ damage markers and cytokines in plasma, liver and spleen. Scatter plots depicting raw levels of cytokines (pg/ul or pg/ug, wherever indicated) used for constructing the heatmap are shown beside the heatmap. Bars represent mean and error bars represent SEM of the mean. All groups were compared to control by two-tailed Mann-Whitney,
****p < 0.0001; ***p < 0.001; **p < 0.01; *p < 0.05; ns, not significant.
The term steroid was used interchangably with glucocorticoid (Gcc), and antibiotic with meropenem (Mem) in all panels of the figure, except the TRRUST network. TRRUST networks from metascape analysis showing transcription factors treatment impact of GccMem8h on kidneys

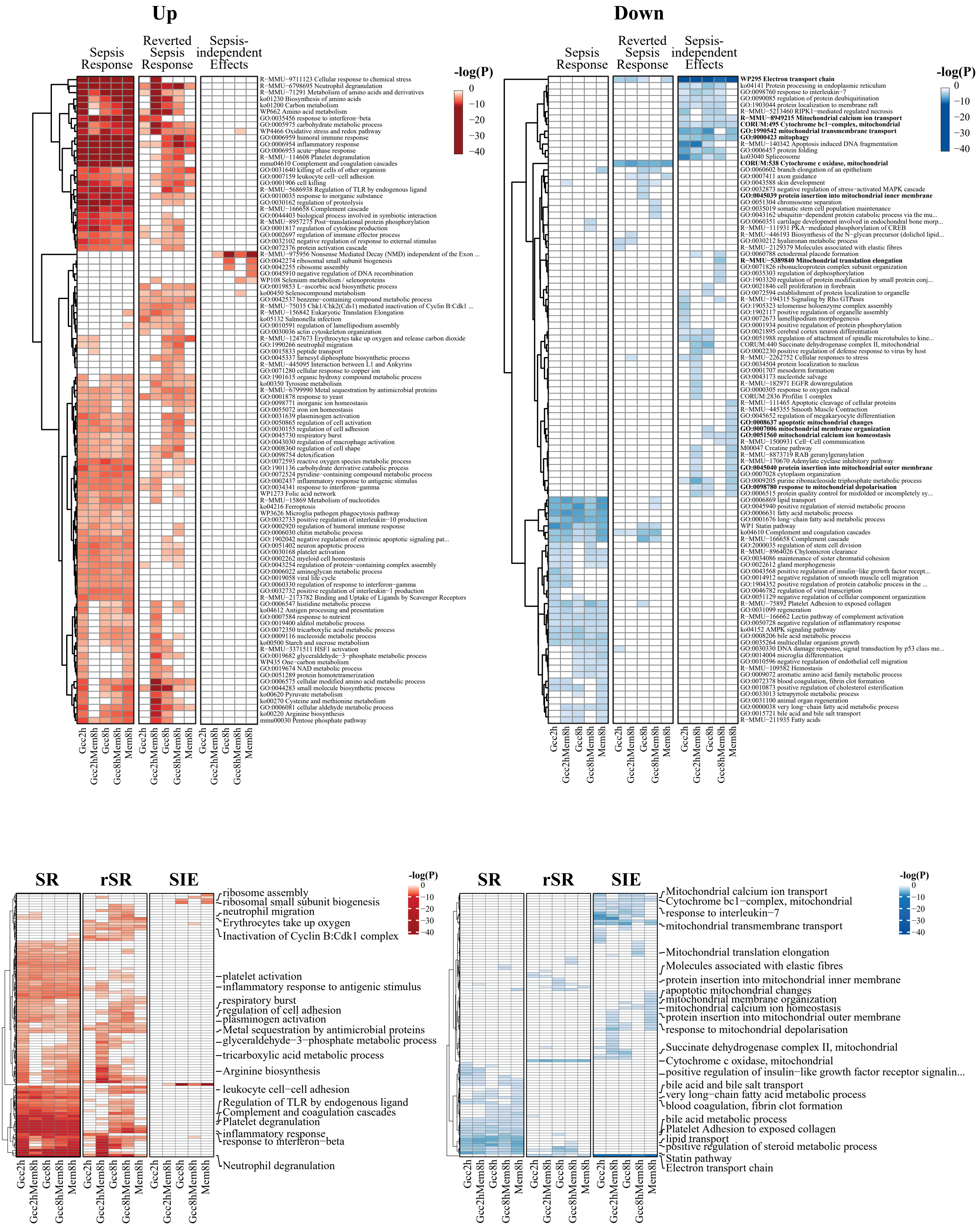

Supplementary Figure 13 Heatmaps depicting the aggregated up- and down-regulated metascape GO terms in different interventions
SR = Sepsis response, rSR = reverted sepsis response and SIE = Sepsis-independent effects

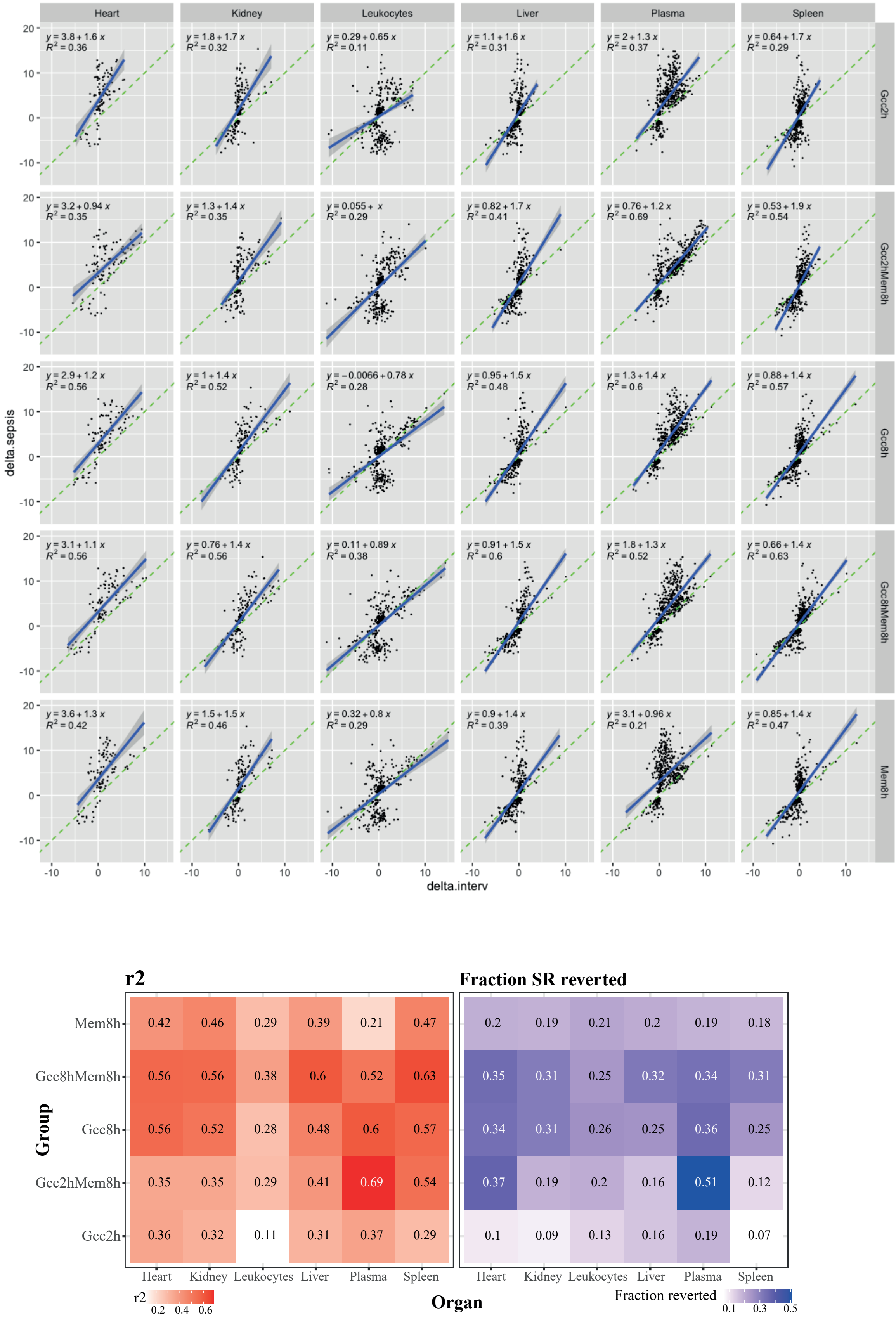

Supplementary Figure 14 Quantification of intervention impact based on the slope and R2 values
Upper panel depicts lots depicteing the slope and R2 values across organs, plasma and leukocytes.
Lower panel depicts a heat map and values only of R2 values and the fraction reverted (Ratio of Reverted DAPs/Total response)
SR reverted = Sepsis response reverted

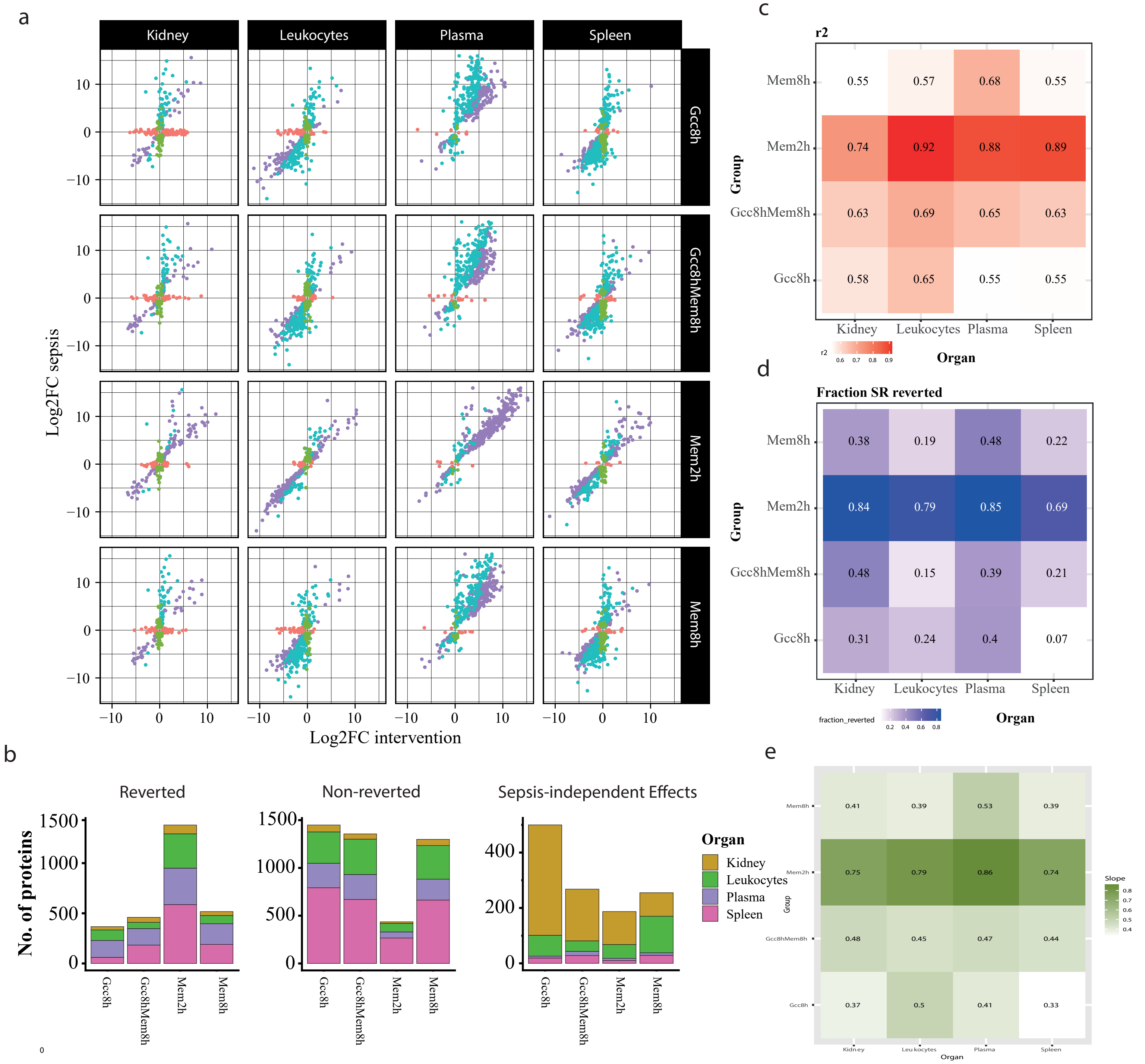

Supplementary Figure 15 Intervention impact as assessed by R2 values and slope in an independent cohort

a) Plots depiciting reversions, non-reversions and side effects in an independent intervention cohort.

b) Number of proteins in organs across reversions, non-reversions and sepsis-independent effects.

c) R2 values depicted as a heatmap

d) Fraction of reversion by interventions

(Fraction SR reverted = Reversions/total response)

e) Heatmap depicting values of the slope of reversion

SR reverted = Sepsis response reverted

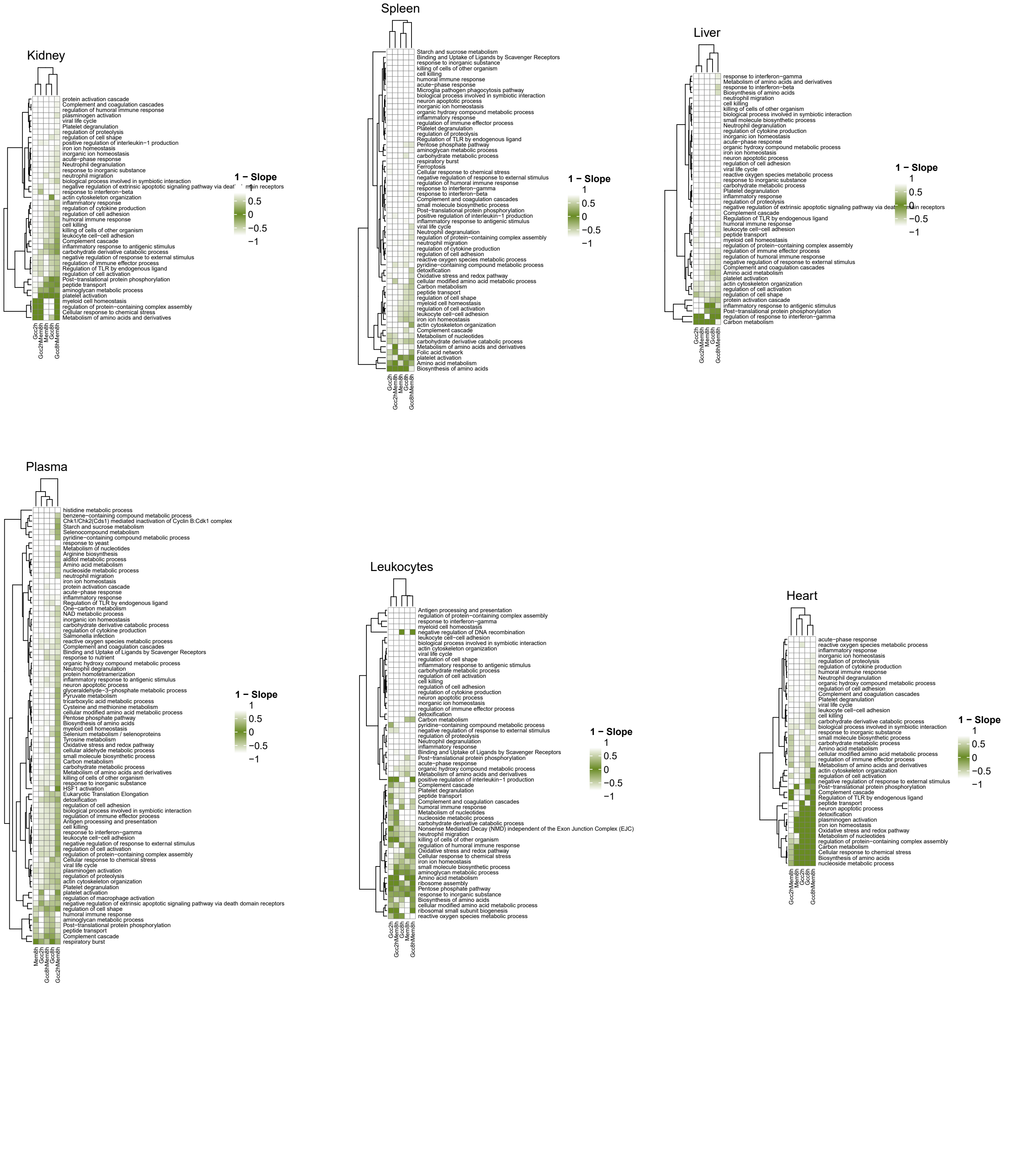

Supplementary Figure 16 Heatmaps showing 1 - Slope for all interventions across all organs
Heatmaps show the level of reversion of proteins belonging to various metascape GO terms by interventions in all organs. A 1-slope value was calculated for each protein category, where protein categories close to 0 are colored indicating the intervention effect per treatment group shown as a heatmap for functional groups associated with increased protein abundance.

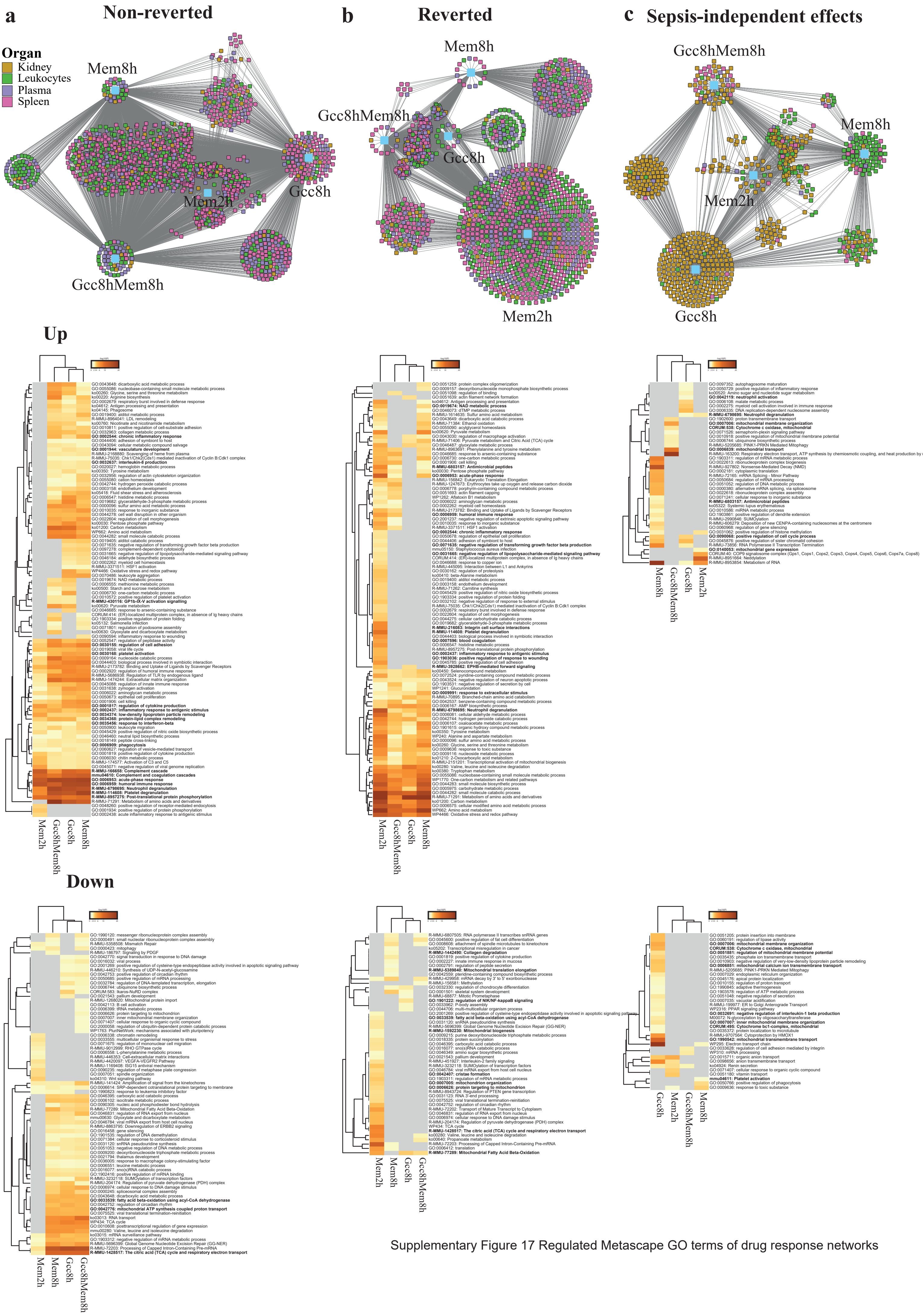

Supplementary Figure 17 Regulated Metascape GO terms of drug response networks

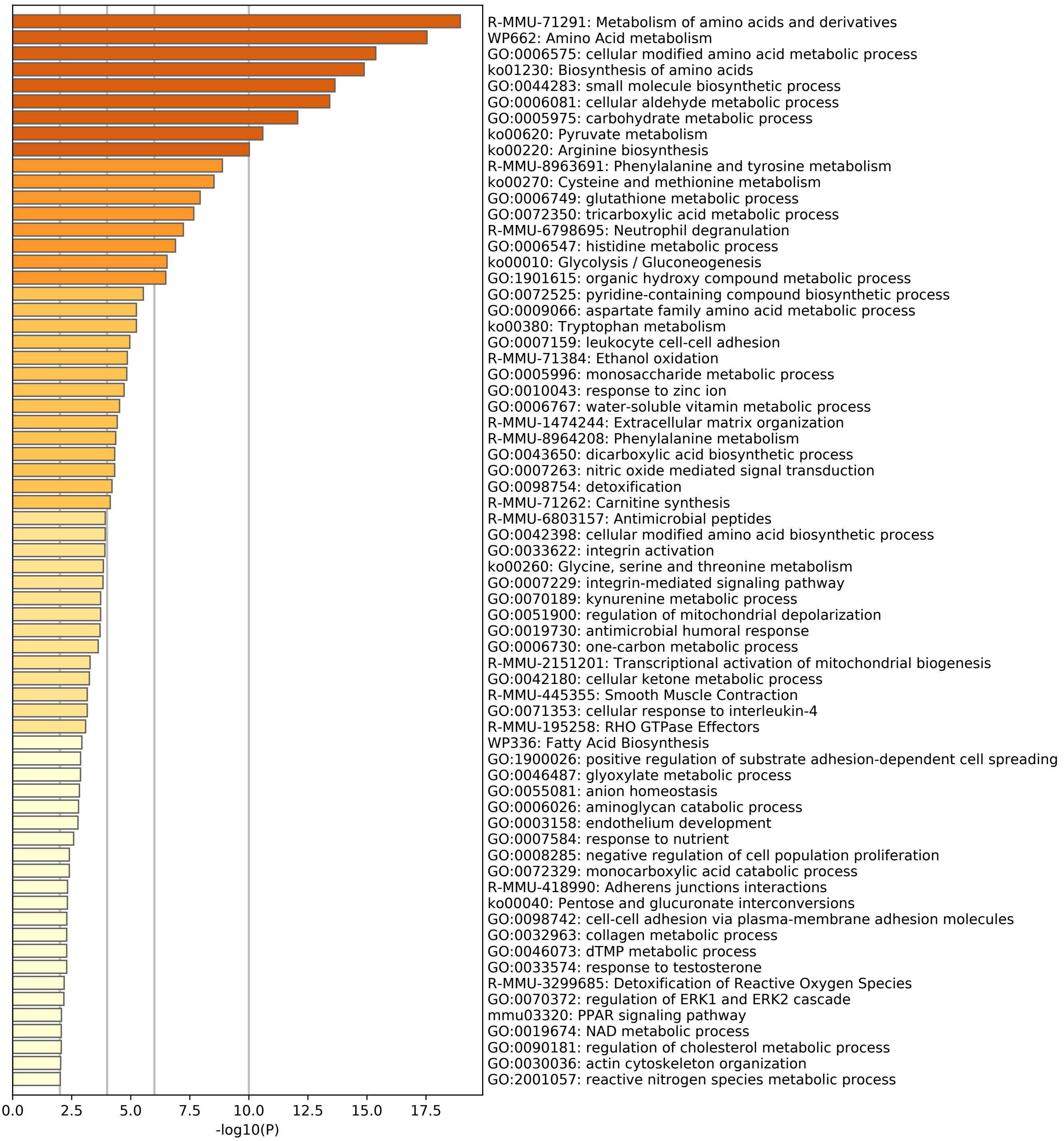

Supplementary Figure 18 - Heatmap depicting metascape GO terms of the 275 tissue enriched leakage proteins in plasma.

Sepsis-independent effects Down

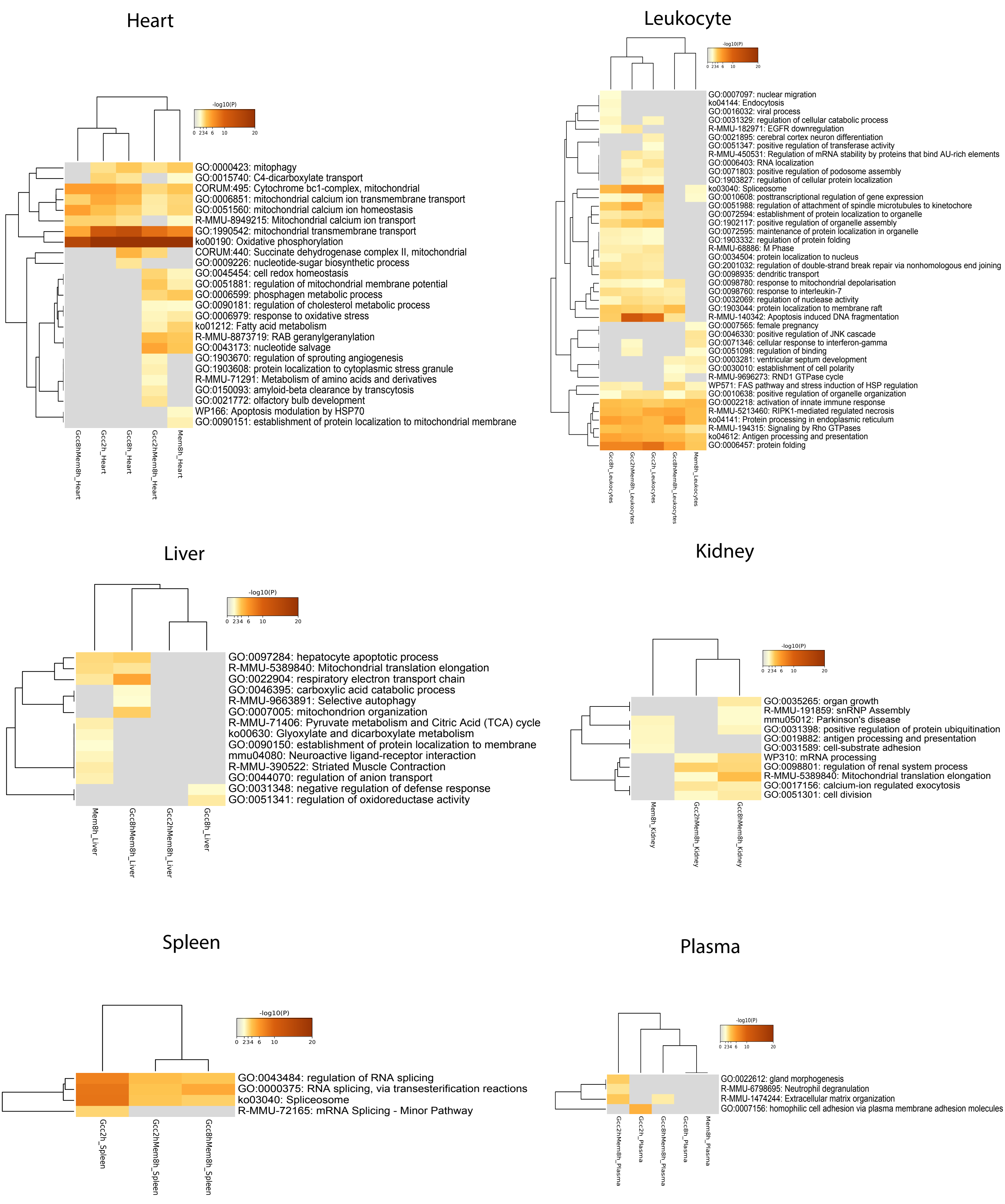

Supplementary Figure 19 Downregulated sepsis-independent effects metascape GO terms

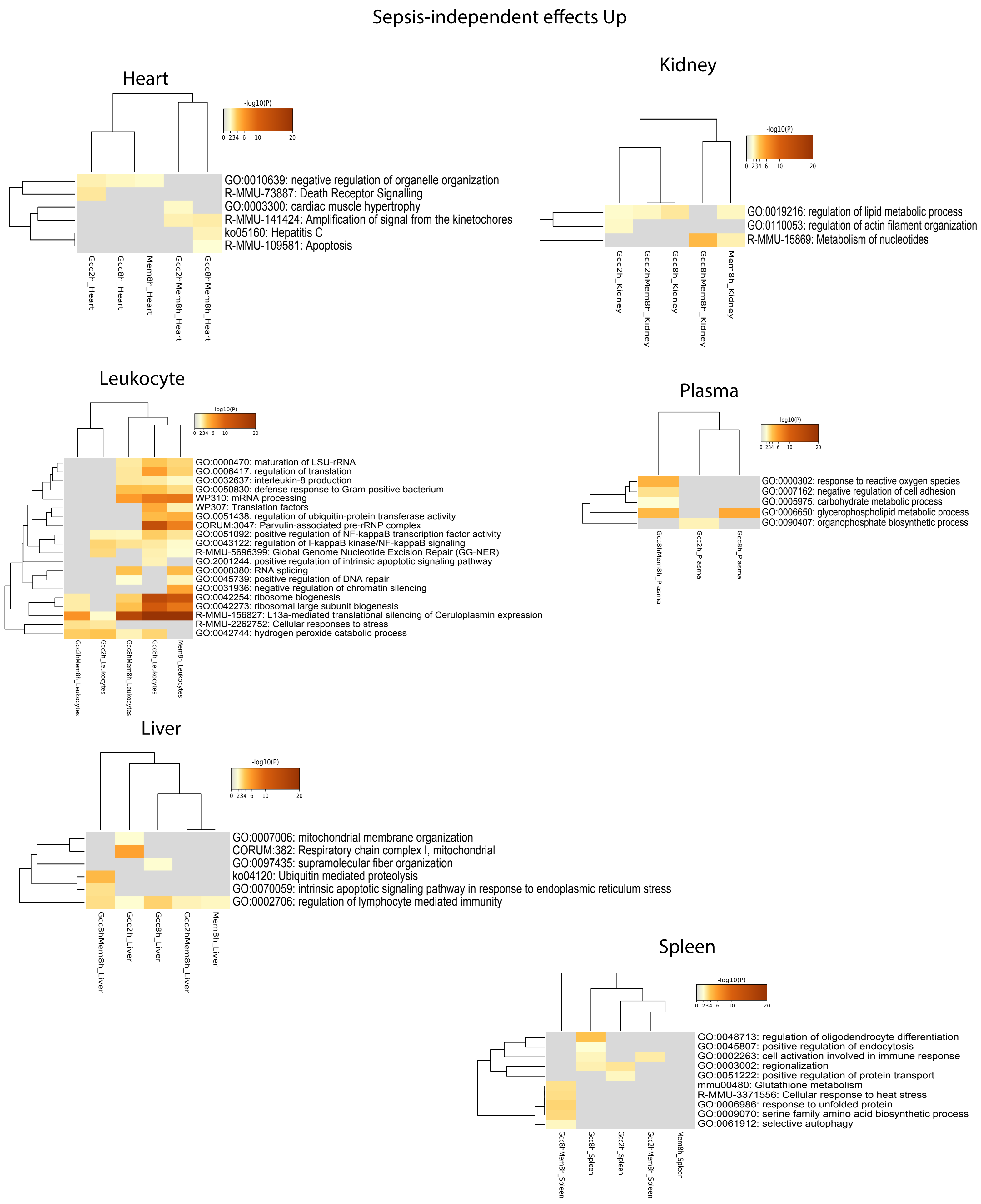

Supplementary Figure 20 Upregulated sepsis-independent effects metascape GO terms

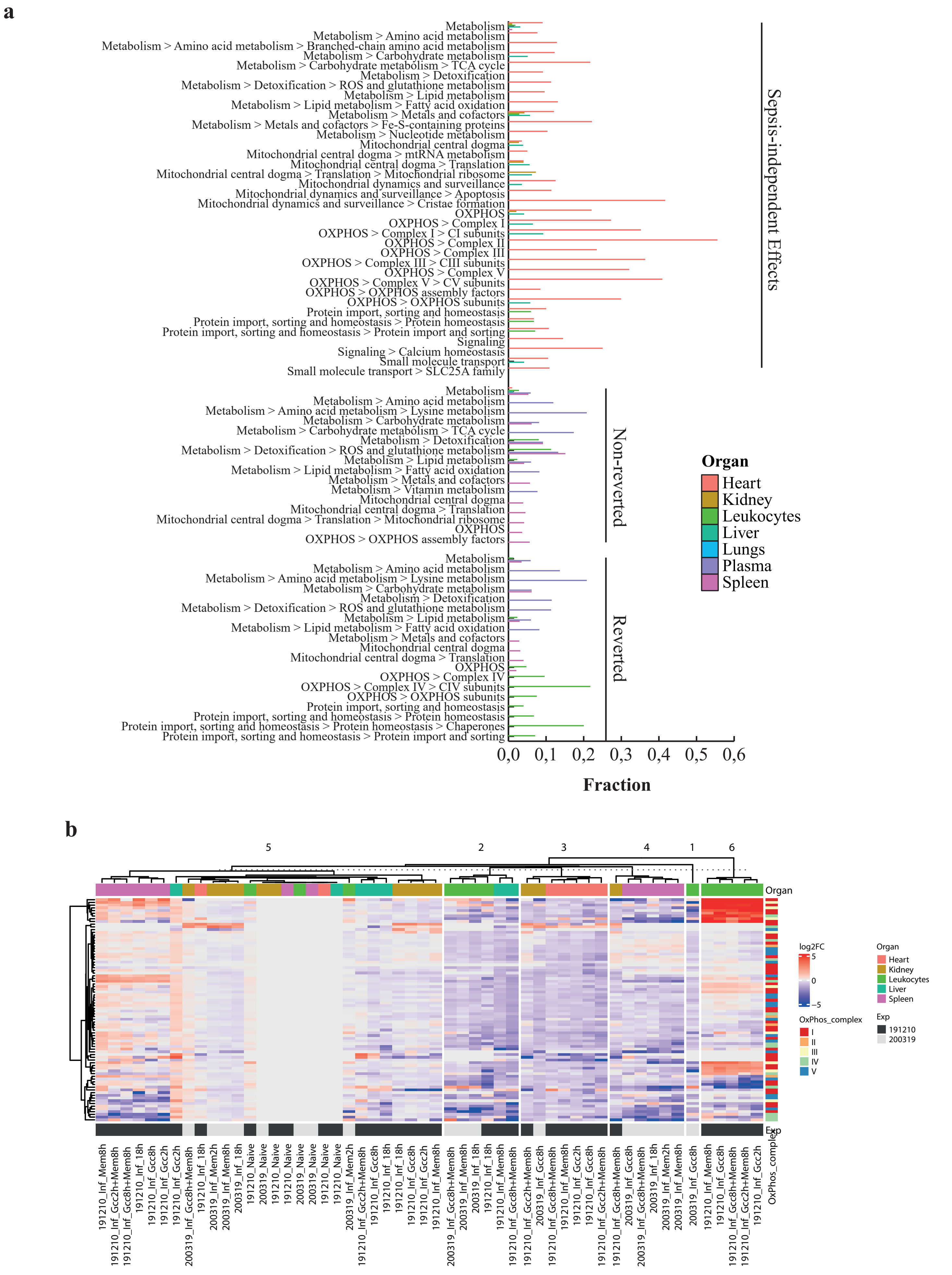

Supplementary Figure 21 Mitocarta enrichment terms in the down regulated sepsis-independent effects (SIE) group

a) Fraction of the proteins found in mitocarta GO terms in the down regulated SIE group

b) Heatmap depicting log2Fc of OXPHOS component across organs

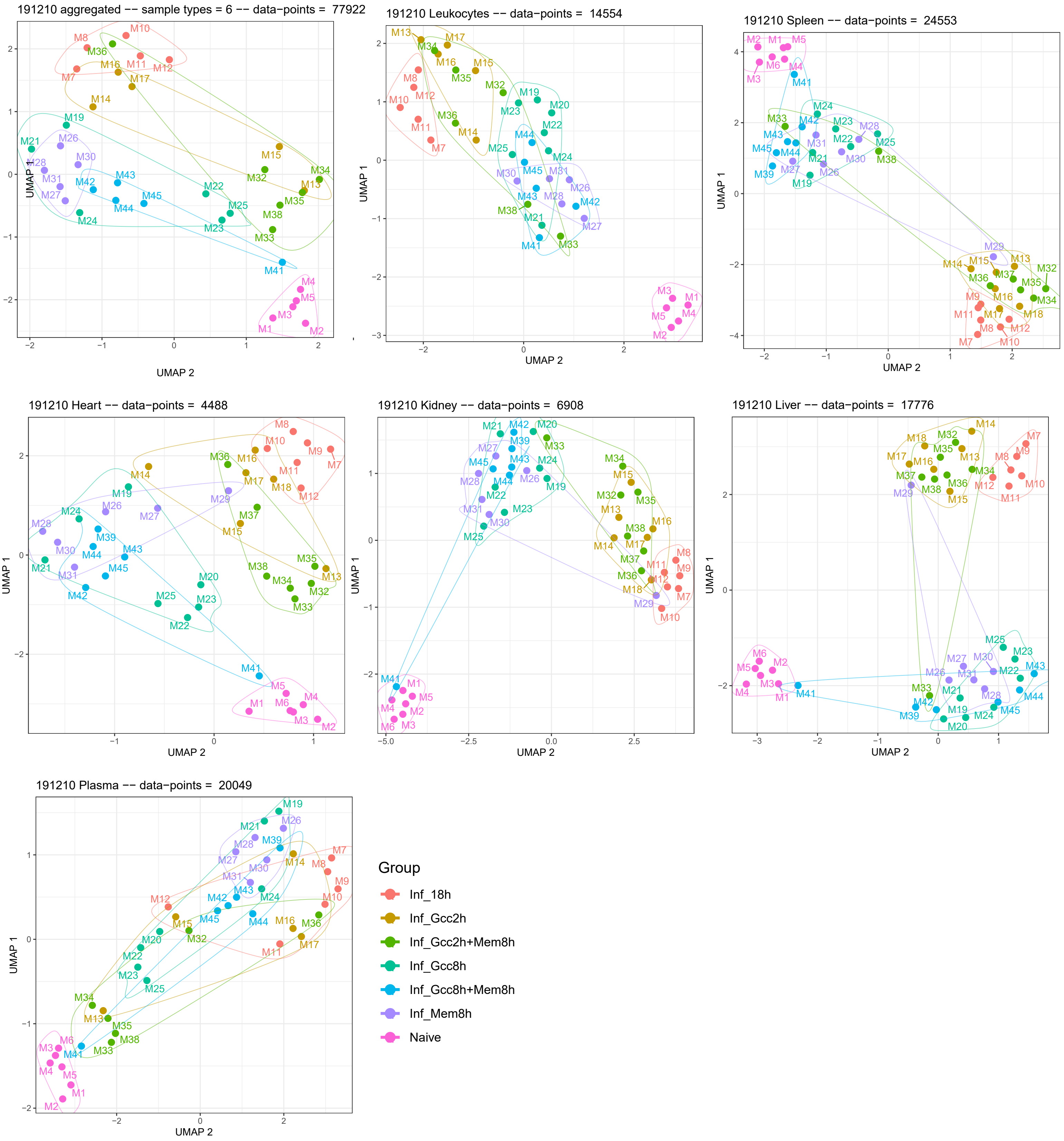

Supplementary Figure 22 Highlighting intervention group clusters using UMAPs.
UMAP visualization was applied to the proteome data from all interventions across all organs and colors indicate tretament groups. Smaller clusters indicate similarity of individuals within an intervention group. Higher spread inicates dissimilar proteome states within an intervention group.

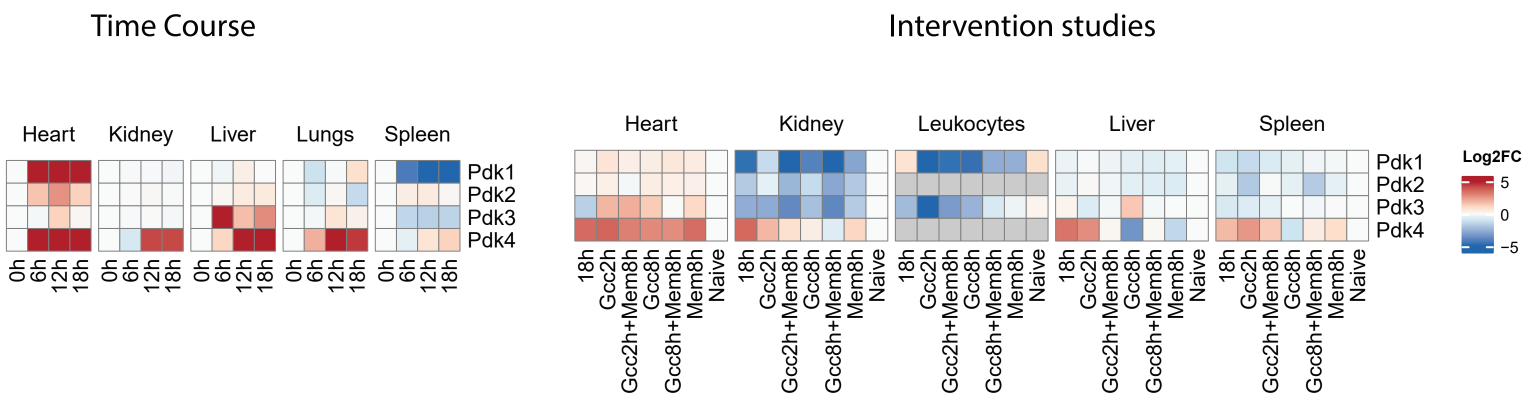

Supplementary Figure 23 Upregulation of pyruvate dehydrogenase kinases (PDKs)
Heatmaps showing the regulation of PDKs. Here we show the levels of the mitochondrial matrix gate keeper enzyme pyruvate dehydrogenase kinase-4 and other isoforms (2-4) in the time course and intervention experiments. Increased PDK4 expression leads to the phosphorylation and inactivation of pyruvate dehydrogenase (PDH) that regulates the entry of pyruvate into the Krebs cycle and oxidative phosphorylation (OXPHOS). A reduction in PDH activity restricts the ability of mitochondria to utilize pyruvate, hence resulting in a lowered oxygen consumption rate. This ensures increased glycolysis and fatty-acid oxidation[1-3]. Despite its very short-lived protein half-life[4], we observed a 6 log-2-fold change in the heart as early as 6 hours and was approximately 5-fold higher than other organs. This early upregulation of PDK4 is likely due to the higher mitochondrial content of the heart. In the treatment cohort, we observed a reversion in the level of PDK4 in the heart and elevation of pyruvate dehydrogenase (PDH) with late Gcc treatments at 8 hours. This combined with the reduced side effects in the mitochondrial OXPHOS components described in figure 6 indicates improved utilization of glucose by mitochondria and a reduction of mitochondrial dysfunction.

1 Kaplon, J. et al. A key role for mitochondrial gatekeeper pyruvate dehydrogenase in oncogene-induced senescence. Nature 498, 109-+, doi:10.1038/nature12154 (2013).
2 Gray, L. R., Tompkins, S. C. & Taylor, E. B. Regulation of pyruvate metabolism and human disease. Cell Mol Life Sci 71, 2577-2604, doi:10.1007/s00018-013-1539-2 (2014).
3 Shimada, B. K. et al. Pyruvate-Driven Oxidative Phosphorylation is Downregulated in Sepsis-Induced Cardiomyopathy: A Study of Mitochondrial Proteome. Shock 57, 553-564, doi:10.1097/Shk.0000000000001858 (2022).
4 Crewe, C., Schafer, C., Lee, I., Kinter, M. & Szweda, L. I. Regulation of Pyruvate Dehydrogenase Kinase 4 in the Heart through Degradation by the Lon Protease in Response to Mitochondrial Substrate Availability. Journal of Biological Chemistry 292, 305-312, doi:10.1074/jbc.M116.754127 (2017).
