# Supplementary material for: A pharmacoproteomic landscape of organotypic intervention responses in Gram-negative sepsis
Source: Nat Commun. 2023 Jun 17;14:3603. doi: 10.1038/s41467-023-39269-9 (PMC10276868; doi:10.1038/s41467-023-39269-9)
Supplement: Supplementary file 3 — Reporting Summary [file 41467_2023_39269_MOESM3_ESM.pdf]

nature portfolio

Corresponding author(s): Johan Malmstrom, Oonagh Shannon

Last updated by author(s): May 5, 2023

Reporting Summary

Nature Portfolio wishes to improve the reproducibility of the work that we publish. This form provides structure for consistency and transparency in reporting. For further information on Nature Portfolio policies, see our Editorial Policies and the Editorial Policy Checklist.

Statistics

For all statistical analyses, confirm that the following items are present in the figure legend, table legend, main text, or Methods section.

n/a	Confirmed	
☐	☒	The exact sample size (*n*) for each experimental group/condition, given as a discrete number and unit of measurement
☐	☒	A statement on whether measurements were taken from distinct samples or whether the same sample was measured repeatedly
☐	☒	The statistical test(s) used AND whether they are one- or two-sided<br>*Only common tests should be described solely by name; describe more complex techniques in the Methods section.*
☒	☐	A description of all covariates tested
☐	☒	A description of any assumptions or corrections, such as tests of normality and adjustment for multiple comparisons
☐	☒	A full description of the statistical parameters including central tendency (e.g. means) or other basic estimates (e.g. regression coefficient) AND variation (e.g. standard deviation) or associated estimates of uncertainty (e.g. confidence intervals)
☐	☒	For null hypothesis testing, the test statistic (e.g. *F*, *t*, *r*) with confidence intervals, effect sizes, degrees of freedom and *P* value noted<br>*Give P values as exact values whenever suitable.*
☒	☐	For Bayesian analysis, information on the choice of priors and Markov chain Monte Carlo settings
☒	☐	For hierarchical and complex designs, identification of the appropriate level for tests and full reporting of outcomes
☒	☐	Estimates of effect sizes (e.g. Cohen's *d*, Pearson's *r*), indicating how they were calculated

Our web collection on statistics for biologists contains articles on many of the points above.

Software and code

Policy information about availability of computer code

Data collection	Flow cytometry data was analyzed using C6 Plus Software (BD Biosciences). All mass spectrometry data was acquired on either a QExactive plus orbitrap or a QExactive HFX orbitrap instrument (Thermoscientific). MS raw data was stored and managed by openBIS (v20.10.0) and converted to centroid indexed mzMLs with ThermoRawFileParser (v1.2.1).

Data analysis

Mass spectrometry data was analyzed with OpenSwathWorkflow (v2.4) and FragPipe (v12.2). The spectral library was generated by searching FragPipe (v12.2), MSFragger (v2.4) and Philosopher (v3.2.3) applying 1% FDR. A spectral library was compiled from the FragPipe output using Spectrast (v5.0), and msproteomicstools (v0.11.0). Decoys were generated with OpenSwathDecoyGenerator v2.4. The OpenSwath data was scored with PyProphet (v2.1.3) utilizing 1% FDR on protein and peptide levels. Finally, the peakgroups were aligned with TRIC from msproteomicstools (v0.11.0). Data analysis was performed using custom scripts in R (3.6) with the R package collection Tidyverse (1.3.0) and Bioconductor package manager BiocManager (v3Statistical analysis was, unless otherwise stated, performed using nonparametric Mann-Whitney tests (Prism v9.1.0 software; GraphPad, Inc). Differential abundance testing was performed with R package limma (1.11.1) or with base R function stats::t.test(). Cut-offs were foldchange > ±1.5 and adjusted (with method of Benjamini, Hochberg) p-value < 0.05. R2 and slope was calculated with R stats::lm. Tissue protein enrichment was calculated the teEnrichmentCustom function with default parameters from the R package TissueEnrich (1.16.0). Functional and pathway enrichment analysis was performed with Metascape (v33) using the web interface (https://metascape.org/) and the 'Express Analysis of Multiple Gene Lists' workflow. Enriched Reactome pathways and Gene Ontology terms (biological processes, cellular components and molecular functions) were determined with R package clusterProfiler (3.4.4) with default parameters. Network analysis graphs of intervention reverted, non-reverted and sepsis-independent effects proteins generated with R package igraph (1.2.6) and visualized with Cytoscape (3.0.0). Heatmaps were generated with R package ComplexHeatmap (v2.0.0) using ward.D2 cluster analysis. Upset plot was generated with R package ComplexHeatmap (v2.15.1) using the default parameters ('distinct mode'). Scripts used for analyses and figure generation of this paper are available at https://doi.org/10.5281/zenodo.7918638).

For manuscripts utilizing custom algorithms or software that are central to the research but not yet described in published literature, software must be made available to editors and reviewers. We strongly encourage code deposition in a community repository (e.g. GitHub). See the Nature Portfolio guidelines for submitting code & software for further information.

Data

Policy information about availability of data

All manuscripts must include a data availability statement. This statement should provide the following information, where applicable:

- Accession codes, unique identifiers, or web links for publicly available datasets
- A description of any restrictions on data availability
- For clinical datasets or third party data, please ensure that the statement adheres to our policy

All proteomics data in the study have been made public. The mass spectrometry proteomics data have been deposited to the ProteomeXchange Consortium via the PRIDE partner repository, PXD036832 (DDA files used in library creation) and PXD036847 (DIA files used for quantification). Functional and pathway enrichment analysis was performed with Metascape (v33, https://doi.org/10.1038/s41467-019-09234-6) using the web interface (https://metascape.org/) and the 'Express Analysis of Multiple Gene Lists' workflow. For each given gene list, pathway and process enrichment analysis have been carried out with the following ontology sources: KEGG Pathway, GO Biological Processes, Reactome Gene Sets, Canonical Pathways, CORUM, WikiPathways, and PANTHER Pathway. The network is visualized using Cytoscape. For each given gene list, protein-protein interaction enrichment analysis has been carried out with the following databases: STRING, BioGrid, OmniPath, InWeb_IM. The Molecular Complex Detection (MCODE) algorithm has been applied to identify densely connected network components. Gene list enrichments are identified in the following ontology categories: Cell_Type_Signatures, DisGeNET, PaGenBase, TRRUST, Transcription_Factor_Targets. All genes in the genome have been used as the enrichment background. Protein sequence database containing the mouse reference proteome (EMBL-EBI RELEASE 2019_04) was used for inferring mouse proteins and MitoCarta3 mouse database was used for defining mitochondrial proteins and subdivision into functional groups.

Human research participants

Policy information about studies involving human research participants and Sex and Gender in Research.

Reporting on sex and gender	NA
Population characteristics	NA
Recruitment	NA
Ethics oversight	NA

Note that full information on the approval of the study protocol must also be provided in the manuscript.

Field-specific reporting

Please select the one below that is the best fit for your research. If you are not sure, read the appropriate sections before making your selection.

☒ Life sciences ☐ Behavioural & social sciences ☐ Ecological, evolutionary & environmental sciences

For a reference copy of the document with all sections, see nature.com/documents/nr-reporting-summary-flat.pdf

Life sciences study design

All studies must disclose on these points even when the disclosure is negative.

Sample size

Sample sizes were chosen based on previously performed pilot experiments and 6-8 animals were included per group. This was sufficient to show differences between various treatment groups or tie points.

Data exclusions	Animals were excluded when the blood volume was too low for flow cytometry.
Replication	The findings were replicated in 2 cohorts for the time course and treatment experiments.
Randomization	Animals were randomized according to the cage housing the animals.
Blinding	The experiments were performed with single-blinding where animals were numerically coded.

Reporting for specific materials, systems and methods

We require information from authors about some types of materials, experimental systems and methods used in many studies. Here, indicate whether each material, system or method listed is relevant to your study. If you are not sure if a list item applies to your research, read the appropriate section before selecting a response.

Materials & experimental systems

n/a	Involved in the study
☐	☒ Antibodies
☒	☐ Eukaryotic cell lines
☒	☐ Palaeontology and archaeology
☐	☒ Animals and other organisms
☒	☐ Clinical data
☒	☐ Dual use research of concern

Methods

n/a	Involved in the study
☒	☐ ChIP-seq
☐	☒ Flow cytometry
☒	☐ MRI-based neuroimaging

Antibodies

Antibodies used

The following antibodies were used in this study:
Alexa Fluor 647 Rat Anti-Mouse CD19, BD Pharmingen, Catalog No. 557684, Clone 1D3
APC-R700 Rat Anti-Mouse Ly-6G Ly-6C, BD Horizon, Catalog No. 565510, Clone RB6-8C5
FITC Mouse Anti-Mouse CD45.2, BD Pharmingen, Catalog No. 553772, Clone 104
FITC Rat Anti-Mouse CD41, BD Pharmingen, Catalog No. 553848, Clone MWReg30
PE Hamster Anti-Mouse CD3e, BD Pharmingen, Catalog No. 553063, Clone 145-2C11
PE Rat Anti-Mouse Ly-6G, BD Pharmingen, Catalog No. 551461, Clone 1A8
PerCP-Cy5.5 Rat Anti-CD11b, BD Pharmingen, Catalog No. 550993, Clone M1/70

Validation

All antibodies were purchased from BD Biosciences. BD Bioscience's website notes that these antibodies are routinely tested for flow cytometry and provides quality certificates for conjugated antibodies certifying that these products have been manufactured and tested in accordance with their specifications (https://www.biocompare.com/Antibody-Manufacturing/355107-Antibody-Manufacturing-Perspectives-BD-Bioscience/). The antibody profiles as listed on the BD website is indicated below as follows -

Alexa Fluor 647 Rat Anti-Mouse CD19, BD Pharmingen, Catalog No. 557684, Clone 1D3 (https://www.bdbiosciences.com/en-nz/products/reagents/flow-cytometry-reagents/research-reagents/single-color-antibodies-ruo/alexa-fluor-647-rat-anti-mouse-cd19.557684)
APC-R700 Rat Anti-Mouse Ly-6G Ly-6C, BD Horizon, Catalog No. 565510, Clone RB6-8C5 (https://www.bdbiosciences.com/en-eu/products/reagents/flow-cytometry-reagents/research-reagents/single-color-antibodies-ruo/APC-R700-Rat-Anti-Mouse-Ly-6G-Ly-6C.565510)
FITC Mouse Anti-Mouse CD45.2, BD Pharmingen, Catalog No. 553772, Clone 104 (https://www.bdbiosciences.com/en-ca/products/reagents/flow-cytometry-reagents/research-reagents/single-color-antibodies-ruo/fitc-mouse-anti-mouse-cd45-2.553772)
FITC Rat Anti-Mouse CD41, BD Pharmingen, Catalog No. 553848, Clone MWReg30 (https://www.bdbiosciences.com/en-ca/products/reagents/flow-cytometry-reagents/research-reagents/single-color-antibodies-ruo/fitc-rat-anti-mouse-cd41.553848)
PE Hamster Anti-Mouse CD3e, BD Pharmingen, Catalog No. 553063, Clone 145-2C11 (https://www.bdbiosciences.com/en-ca/products/reagents/flow-cytometry-reagents/research-reagents/single-color-antibodies-ruo/pe-hamster-anti-mouse-cd3e.553063)
PE Rat Anti-Mouse Ly-6G, BD Pharmingen, Catalog No. 551461, Clone 1A8 (https://www.bdbiosciences.com/en-ca/products/reagents/flow-cytometry-reagents/research-reagents/single-color-antibodies-ruo/pe-rat-anti-mouse-ly-6g.551461)
PerCP-Cy5.5 Rat Anti-CD11b, BD Pharmingen, Catalog No. 550993, Clone M1/70 (https://www.bdbiosciences.com/en-ca/products/reagents/flow-cytometry-reagents/research-reagents/single-color-antibodies-ruo/percp-cy-5-5-rat-anti-cd11b.550993)

Animals and other research organisms

Policy information about studies involving animals; ARRIVE guidelines recommended for reporting animal research, and Sex and Gender in Research

Laboratory animals

Nine-week-old female and male C57BL/6J mice (Janvier, Le Genest-Saint-Isle, France). The animals were maintained in a facility with 12 light/12 dark cycle with an ambient temperature of approximately 20°C. Technicians and staff did not enter the room during the dark cycle unless strictly required to collect cages for health monitoring.

Wild animals	No wild animals were used in the study.
Reporting on sex	Age and sex matched groups were used for the study.
Field-collected samples	No field collected samples were used in the study.
Ethics oversight	All animal use and procedures were approved by the local Malmö/Lund Institutional Animal Care and Use Committee, ethical permit number 03681-2019.

Note that full information on the approval of the study protocol must also be provided in the manuscript.

Flow Cytometry

Plots

Confirm that:

☒ The axis labels state the marker and fluorochrome used (e.g. CD4-FITC).

☒ The axis scales are clearly visible. Include numbers along axes only for bottom left plot of group (a 'group' is an analysis of identical markers).

☒ All plots are contour plots with outliers or pseudocolor plots.

☒ A numerical value for number of cells or percentage (with statistics) is provided.

Methodology

Sample preparation	For the treatment cohort, counts for WBCs, neutrophils, monocytes, and platelets were determined by volumetric flow cytometry. Citrated blood collected from infected and control mice was diluted with HEPES buffer containing mouse Fc-block (BD Pharmingen). The samples were stained with antibodies (1:200) and incubated for 15 min at room temperature. Samples were then lysed using 1-step Fix/Lyse Solution (e-Bioscience) for 30 min at room temperature, washed with PBS (500 rcf, 5 min), and the cell pellets were resuspended in PBS.
Instrument	The samples were analyzed using an Accuri C6 Plus flow cytometer (BD Biosciences)
Software	The data was analyzed using C6 Plus Software (BD Biosciences)
Cell population abundance	No Sorting of cells was performed. Hence, not applicable.
Gating strategy	Two flow cytometry panels were used. In the first panel, platelets were gated according to forward scatter and CD61. Total leukocytes were gated (R1) according to characteristic forward and side scatter. From total leukocytes (R1 gate), neutrophils were gated as Ly-6G and Ly-6C high. The MFI of CD11b in the neutrophil gate was determined. Lmphocytes and monocytes were gated together as Ly-6G low and Ly-6C intermediate/high. Monocytes were distinguished from lymphocytes with CD11b. In the second panel, CD45.2-positive leukocytes were gated from total leukocytes (R1). From CD45.2-leukocytes, T cells were identified with CD3e and B cells were identified with CD19.

☒ Tick this box to confirm that a figure exemplifying the gating strategy is provided in the Supplementary Information.
